# Microscale 3D Liver Bioreactor for In Vitro Hepatotoxicity Testing under Perfusion Conditions

**DOI:** 10.3390/bioengineering5010024

**Published:** 2018-03-15

**Authors:** Nora Freyer, Selina Greuel, Fanny Knöspel, Florian Gerstmann, Lisa Storch, Georg Damm, Daniel Seehofer, Jennifer Foster Harris, Rashi Iyer, Frank Schubert, Katrin Zeilinger

**Affiliations:** 1 Berlin-Brandenburg Center for Regenerative Therapies (BCRT), Charité–Universitätsmedizin Berlin, 13353 Berlin, Germany; selina.greuel@charite.de (S.G.); fanny.knoespel@gmx.de (F.K.); f.gerstmann@mailbox.tu-berlin.de (F.G.); lisa.storch@gmx.net (L.S.); katrin.zeilinger@charite.de (K.Z.); 2 Department of Hepatobiliary Surgery and Visceral Transplantation, University of Leipzig, 04103 Leipzig, Germany; georg.damm@medizin.uni-leipzig.de (G.D.); daniel.seehofer@medizin.uni-leipzig.de (D.S.); 3 Los Alamos National Laboratory, Los Alamos, NM 87545, USA; jfharris@lanl.gov (J.F.H.); rashi@lanl.gov (R.I.); 4 StemCell Systems GmbH, Berlin 12101, Germany; frank.schubert@stemcell-systems.com

**Keywords:** microscale 3D liver bioreactor, in vitro perfusion, primary human liver cells, hepatotoxicity, acetaminophen

## Abstract

The accurate prediction of hepatotoxicity demands validated human in vitro models that can close the gap between preclinical animal studies and clinical trials. In this study we investigated the response of primary human liver cells to toxic drug exposure in a perfused microscale 3D liver bioreactor. The cellularized bioreactors were treated with 5, 10, or 30 mM acetaminophen (APAP) used as a reference substance. Lactate production significantly decreased upon treatment with 30 mM APAP (*p* < 0.05) and ammonia release significantly increased in bioreactors treated with 10 or 30 mM APAP (*p* < 0.0001), indicating APAP-induced dose-dependent toxicity. The release of prostaglandin E2 showed a significant increase at 30 mM APAP (*p* < 0.05), suggesting an inflammatory reaction towards enhanced cellular stress. The expression of genes involved in drug metabolism, antioxidant reactions, urea synthesis, and apoptosis was differentially influenced by APAP exposure. Histological examinations revealed that primary human liver cells in untreated control bioreactors were reorganized in tissue-like cell aggregates. These aggregates were partly disintegrated upon APAP treatment, lacking expression of hepatocyte-specific proteins and transporters. In conclusion, our results validate the suitability of the microscale 3D liver bioreactor to detect hepatotoxic effects of drugs in vitro under perfusion conditions.

## 1. Introduction

The evaluation of hepatotoxicity of pharmaceutical substances is one major aspect of drug development. For in vivo hepatotoxicity testing, animal models, especially rats and mice, are currently the method of choice [1,2]. However, the use of such models is controversial as they often do not accurately represent the human metabolism due to differences in pharmacokinetics, pharmacodynamics, and species-specific genetic variations [3,4]. The accurate prediction of potential hepatotoxicity demands validated human in vitro models that can close the gap between preclinical animal studies and clinical trials in drug toxicity testing.

Currently applied in vitro toxicity testing models, especially in earlier developmental stages, are mostly based on 2D cell cultures, which offer several advantages including low costs, high throughput, and reproducibility. However, conventional 2D models using primary human hepatocytes are impeded by a rapid decrease of hepatic function and by cell dedifferentiation [5]. This phenomenon is partly due to loss of the original 3D architecture of the organ, which is characterized by organ-specific cell–cell and cell–extracellular matrix contacts. A promising approach to create a physiologically relevant surrounding in vitro can be seen in the development of 3D culture models [6]. Evidence shows that 3D models better reflect the microcellular environment than 2D cultures and thereby enable a more realistic prediction of in vivo drug effects [7,8,9]. Moreover, an even closer approximation of the in vivo situation is provided by perfused culture platforms that mimic the in vivo hemodynamics and enhance nutrient supply of the cells [10,11]. In addition, perfused culture models enable a constant exposure to test compounds with simultaneous removal of metabolites, in contrast to static 2D cultures with discontinuous medium exchange. In this context, microfluidic culture systems gain increasing importance, since they allow minimization of the amounts of cells and reagents needed while providing characteristics of 3D cultures with physiological cell arrangement [12,13].

We have previously shown that a scalable 3D multicompartment bioreactor technology with counter-current medium exchange and decentralized oxygenation supports the reorganization and longevity of primary human liver cells in vitro [14]. It was also demonstrated that the technology is suitable for analysis of hepatic drug metabolism and hepatic toxicity using serum-free conditions [15,16,17]. Based on the existing technology, a microscale 3D liver bioreactor with a cell compartment volume of 100 µL was constructed for applications in preclinical drug development and toxicity testing.

The goal of the present study was to investigate the potential of the device to detect toxic drug effects, using acetaminophen (APAP) as a reference substance. For this purpose, primary human liver cells were cultured in microscale 3D bioreactors over six days and treated with APAP at final concentrations of 0, 5, 10, or 30 mM. For monitoring the cell viability and functionality, the release of intracellular enzymes, parameters of glucose and nitrogen metabolism, as well as the liberation of inflammatory factors were measured daily. Upon termination of the bioreactor cultures, histological and immunofluorescence as well as mRNA analysis were performed to detect possible effects of APAP on the tissue integrity and gene expression of hepatic markers.

## 2. Materials and Methods

### 2.1. Bioreactor System

**Figure 1:**
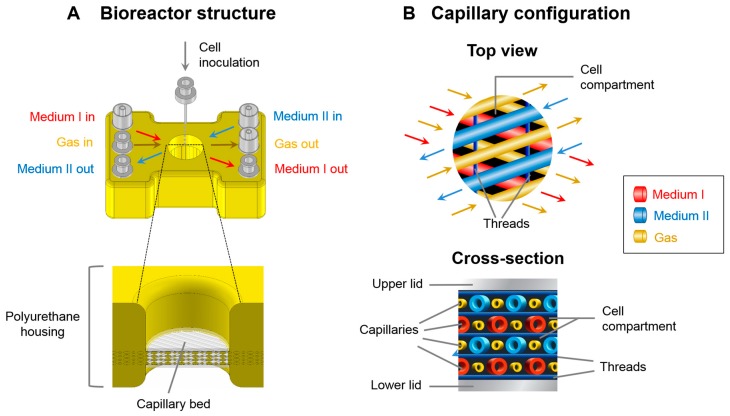
Schematic illustration of the structure of the microscale 3D liver bioreactor. (**A**) Outside view of the bioreactor with tube connections for medium in- and outflow via two independent capillary systems (Medium I and Medium II), perfusion with an air/CO_2_ mixture (Gas), and a tube serving for cell inoculation into the extracapillary space (cell compartment); the figure below shows a section of the bioreactor housing with a central cavity containing the capillary bed; (**B**) Capillary structure of the bioreactor shown as top view (upper figure) and cross-section (lower figure). The capillary bed consists of four layers of hollow-fiber capillaries. Cells are seeded in the extra-capillary space (cell compartment). The layers form a 45° angle to each other to enable counter-current medium perfusion of the two capillary systems (red: Medium I, blue: Medium II). Synthetic threads (dark blue) integrated between each layer serve as spacers.

The microscale 3D bioreactor used in this study is based on a four-compartment hollow-fiber bioreactor technology described previously [15,17] and was further down-scaled resulting in a culture volume of about 100 µL for perfusion cell culture at microscale level. Figure 1 shows the bioreactor structure and the configuration of hollow-fiber capillaries in the device. The bioreactor housing (Figure 1A) is made of polyurethane and sized in credit card format. It disposes of tube connections for medium perfusion via two independent capillary systems (Medium I and Medium II), perfusion with an air/CO_2_ mixture (Gas), and cell inoculation. A central cavity scaled at 1 cm in diameter harbors the capillary bed, which is made of four hollow-fiber layers. As shown in Figure 1B, each layer is composed of alternately arranged medium and gas capillaries, which serve for cell nutrition and oxygenation while providing an adhesion scaffold for the cells seeded in the extra-capillary space (cell compartment). The arrangement of capillary layers in a 45° angle to each other allows for counter-current medium perfusion of the capillary bed. Mass exchange between the capillary lumen and the cell compartment occurs via the pores of the used hydrophilic high-flux filtration membranes (3M, Neuss, Germany), while air/CO_2_ exchange is mediated via hydrophobic membranes (Mitsubishi, Tokyo, Japan).

Synthetic threads made of polyethylenterephtalate are placed as spacers between the capillary layers. Thus, direct contacts between the capillaries, which could result in shunt formation and impaired mass exchange, are prevented.

**Figure 2:**
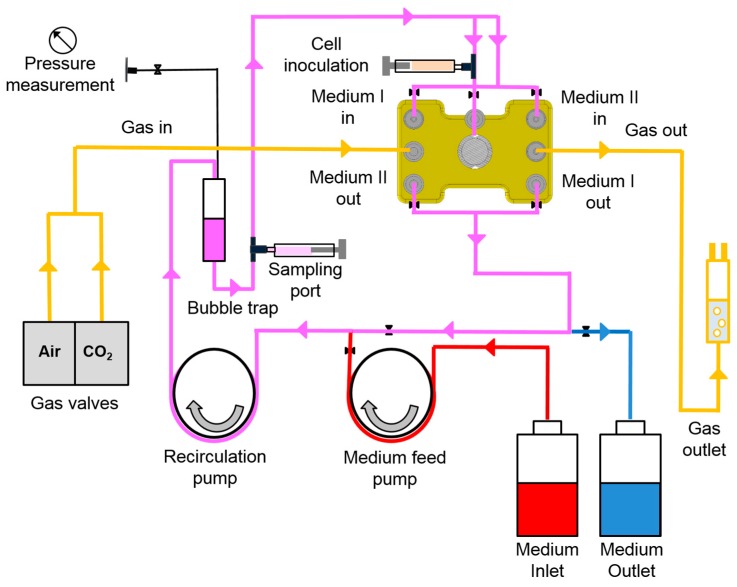
Schematic illustration of the bioreactor perfusion circuit. The bioreactor is integrated into a tubing circuit with continuously recirculating medium (purple). Fresh medium (red) is infused into the circuit via the medium feed pump while used medium (blue) is rinsed out from the circuit upon hydrostatic pressure increase. The tubing circuit contains a bubble trap connected with a line for pressure measurement and is equipped with connections for sample taking. Furthermore, the bioreactor is perfused with a defined air/CO_2_ mixture (yellow), which is generated by means of electronically controlled gas valves.

Bioreactors are run in a perfusion circuit consisting of tubing for medium recirculation, medium feed, and medium outflow, as well as gas perfusion lines, as shown schematically in Figure 2. Medium flow rates are regulated by individual pumps for medium recirculation and medium feed, while electronically controlled gas valves (Vögtlin Instruments, Aesch, Switzerland) serve for regulation of air and CO_2_ flow rates. The temperature in the bioreactor chamber is maintained at a constant level by means of software-controlled heating cartridges (HS Heizelemente GmbH, Fridingen, Germany).

### 2.2. Primary Human Liver Cell Isolation

Primary human liver cells were gained from tissue remaining from clinical partial liver resection. All patients gave their informed written consent before they participated in the study. The study was conducted in accordance with the Declaration of Helsinki, and the protocol was approved by the Ethics Committee of the Charité–Universitätsmedizin Berlin (EA2/026/09, dated 11 April 2013). Cell isolation was performed by collagenase digestion according to previously described protocols [18,19]. The obtained cell suspension was not further purified to avoid a loss of non-parenchymal cell fractions. The cell viability after isolation was 84.6 ± 7.0% as determined by their capacity to exclude trypan blue.

### 2.3. Bioreactor Operation

Primary human liver cells were seeded into each bioreactor at 10^7^ cells per bioreactor. Liver cell bioreactors were perfused with Heparmed culture medium (Vito 143, Biochrom, Berlin, Germany), a modification of Williams’ Medium E specifically developed for serum-free perfusion of high-density 3D liver cell cultures. The medium was supplemented prior to use with 20 IU/L insulin, 5 mg/L transferrin, 3 µg/L glucagon, 100,000 U/L penicillin, and 100 mg/L streptomycin (all purchased from Biochrom). Culture medium recirculated at a rate of 1 mL/min, while fresh medium was continuously fed into the perfusion circuit at a rate of 0.6 mL/h for the first 24 h followed by 0.2 mL/h until the end of culture. Used medium was removed at the same rate and flowed into the outlet vessel. The pH value in the recirculating medium was kept between 7.35 and 7.45 by adjusting the CO_2_ concentration in the supplied gas mixture. Perfusion parameters and operation conditions for running the microscale bioreactors are listed in Table 1.

### 2.4. Clinical Chemistry Parameters

Parameters for assessment of the cell viability and functionality in the bioreactors were measured daily in samples from the culture perfusate. Activities of intracellular enzymes, including lactate dehydrogenase (LDH), aspartate aminotransferase (AST), alanine transaminase (ALT), and glutamate dehydrogenase (GLDH), as well as urea and ammonia concentrations were measured at Labor Berlin GmbH, Berlin, Germany, using automated clinical chemistry analyzers (Cobas^®^ 8000, Roche Diagnostics GmbH, Mannheim, Germany). Glucose and lactate levels were analyzed by means of a blood gas analyzer (ABL 700, Radiometer, Copenhagen, Denmark). Concentrations of prostaglandin E2 (PGE2; Life Technologies, Carlsbad, CA, USA) and interleukin-6 (IL-6; Peprotech, Rocky Hill, NJ, USA) were determined using ELISA Kits according to the instructions of the manufacturers. The required sample volumes and variation coefficients are provided in Table 2.

### 2.5. Acetaminophen (APAP) Application

On the third day of culture, APAP (Sigma-Aldrich, St.-Louis, MO, USA) was added at a final concentration of 5, 10, or 30 mM. The drug was dissolved in methanol, followed by methanol evaporation and dissolution of the substance in culture medium. APAP incubation was initiated by adding 1 mL of a 7× concentrated solution of the drug into the perfusion circuit via the bubble trap (bolus application), to reach the desired final solution of the drug in the recirculation circuit. Subsequently, fresh medium containing APAP at the respective concentration of 5, 10, or 30 mM was continuously infused into the perfusion circuit. The control bioreactor was treated equally, but without adding the drug.

### 2.6. Histological and Immunofluorescence Analysis

Upon termination of bioreactor cultures on day 6 of culture, bioreactors were opened and sections of the capillary bed containing the cell material were taken. Histological slides were prepared from fixed samples and subjected to hematoxylin-eosin (HE) and immunofluorescence staining as described previously [15]. Double-staining of antigens was performed using monoclonal mouse anti-cytochrome P450 (CYP) 1A2 antibodies (AB) provided by Santa Cruz (Santa Cruz, CA, USA), combined with monoclonal rabbit anti-cytokeratin 18 (CK18) AB (Abcam, Cambridge, UK); monoclonal mouse anti-CK18 AB (Santa Cruz), combined with polyclonal rabbit anti-vimentin AB (Santa Cruz); or monoclonal mouse anti-multidrug resistance protein 2 (MRP2) AB (Abcam) in combination with polyclonal rabbit anti-CYP3A4 AB (Abcam). As secondary AB, fluorochrome-coupled goat anti-mouse IgG 488 AB (Life Technologies) and goat anti-rabbit IgG 594 AB (Life Technologies) were used. Counterstaining of nuclei was performed using bisbenzimide H 33342 trihydrochloride (Hoechst 33342, Sigma-Aldrich).

### 2.7. qRT-PCR

Total RNA was obtained from human liver cells gained from the bioreactors after termination of the cultures on day 6. The RNA was extracted using the TRIzol^®^ Reagent (Life Technologies) according to the manufacturer’s instructions. Afterwards, genomic DNA was digested using the RNase-free DNase-Set (Qiagen, Hilden, Germany). Subsequent cDNA synthesis and quantitative real-time PCR (qRT-PCR) were performed as described elsewhere [20] using human-specific primers and probes (TaqMan Gene Expression Assay system, Life Technologies, Table 3). The expression of specific genes was normalized to that of the housekeeping gene glyceraldehyde-3-phosphate dehydrogenase (*GAPDH*) and fold changes of expression levels were calculated with the ΔΔ*C*_t_ method [21].

### 2.8. Statistics

Four independent experiments were performed with cells from different donors (*N* = 4, unless stated otherwise). Statistical analyses were performed using GraphPad Prism 5.0 for Windows (GraphPad Software, San Diego, CA, USA). Results are provided as mean ± standard error of the mean (SEM). The influence of the drug dose (day 3–day 6) on clinical chemistry parameters in comparison to the control was analyzed by calculating the area under curve (AUC) of values during the drug application interval. The AUCs between day 3 and day 6 of the groups treated with different APAP concentrations were compared with those of untreated control cultures by means of one-way ANOVA with Dunnett’s multiple comparison test. The same test was used for statistical evaluation of gene expression data. The group treated with 30 mM APAP was not included in the statistical analysis of gene expression data, since RNA in sufficient quality and quantity was only gained from one culture in this group.

## 3. Results

### 3.1. Clinical Chemistry Parameters

**Figure 3:**
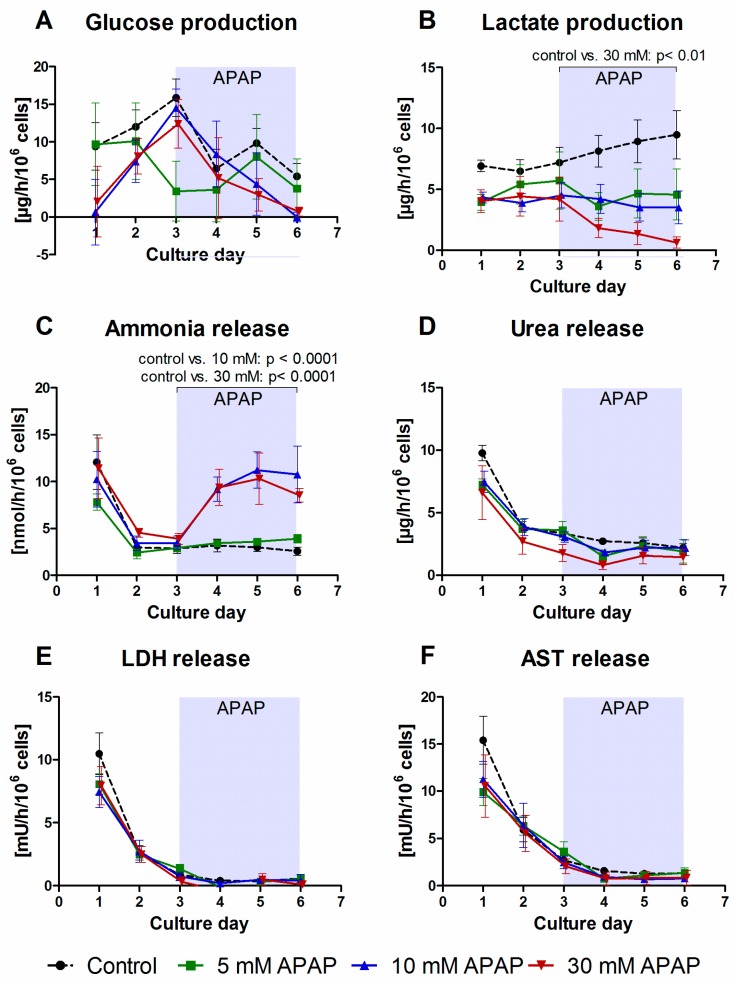
Time-courses of clinical parameters in bioreactors treated with 5 mM, 10 mM or 30 mM acetaminophen (APAP) in comparison to untreated bioreactors used as control group. The figure shows the course of glucose (**A**) and lactate (**B**) production, ammonia (**C**) and urea (**D**) release, as well as liberation of lactate dehydrogenase (LDH, (**E**)) and aspartate aminotransferase (AST, (**F**)). APAP was continuously introduced from day 3 throughout day 6 of culture. Values were normalized to 10^6^ inoculated cells. Data are shown as means ± SEM (*n* = 4; control *n* = 6). The influence of the drug dose (day 3–day 6) on the metabolic activity of the cells in comparison to the control was analyzed by means of one-way ANOVA with Dunnett’s multiple comparison test, using the AUCs from day 3 until day 6. Significant changes are indicated in the graphs. Underlying data are available at http://doi.org/10.5281/zenodo.1169306 (Clinical_chemistry_parameters).

Clinical chemistry parameters revealed a dose-dependent effect of APAP on metabolic functions of primary human liver cells maintained in perfused microscale bioreactors (Figure 3).

The time-course of glucose production (Figure 3A) showed stable values with some fluctuations in control bioreactors or those treated with 5 mM APAP, while a clear decrease upon drug application from day 3 onwards was observed in bioreactors exposed to 10 or 30 mM APAP. Lactate production rates (Figure 3B) showed a steadily increasing course in the control group, while bioreactors exposed to 5 or 10 mM APAP remained on a constant level, and the group exposed to 30 mM APAP was characterized by a sharp decline. The comparison of AUCs of lactate values following drug application revealed a significant difference between the 30 mM APAP-treated group and the control group (*p* < 0.01).

The time-course of ammonia release was determined as an indicator for the cells’ capacity of nitrogen elimination (Figure 3C). After an initial peak on the first culture day, control bioreactors and those treated with 5 mM APAP showed stable values on a basal level. In contrast, a distinct increase was observed in bioreactors upon exposure to 10 or 30 mM APAP, with significantly (*p* < 0.0001) increased AUCs as compared with the control group. Urea production rates showed a mild, but not significant decrease at 30 mM APAP, while lower drug concentrations did not affect urea levels as compared to untreated control bioreactors (Figure 3D).

Release rates of the intracellular enzymes LDH and AST, indicating disturbed cell integrity and membrane leakage, showed a similar time-course in all experimental groups, characterized by a peak immediately after cell inoculation, which was followed by basal levels from day 3 onwards (Figure 3E,F). The enzymes ALT and GLDH showed a similar time course (data available at http://doi.org/10.5281/zenodo.1169306 (clinical chemistry parameters)). The administration of APAP had no effect on enzyme release rates.

**Figure 4:**
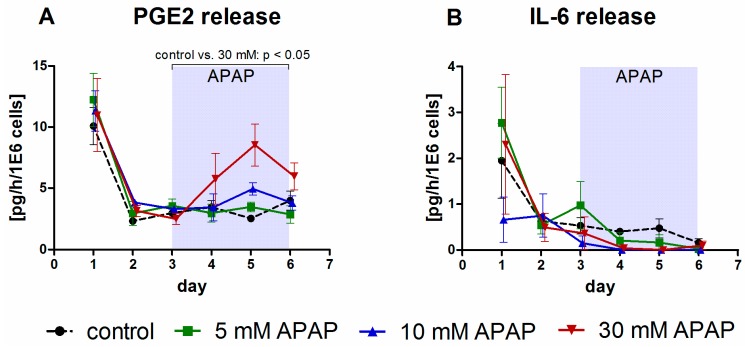
Time-courses of inflammatory factors in bioreactors treated with 5 mM, 10 mM, or 30 mM acetaminophen (APAP) in comparison to untreated bioreactors used as control group. The figure shows release rates of prostaglandin E2 (PGE2, (**A**)) and interleukin 6 (IL-6, (**B**)). APAP was continuously introduced from day 3 throughout day 6 of culture. Values were normalized to 10^6^ inoculated cells. Data are shown as means ± SEM (*n* = 3; control *n* = 5). The influence of the drug dose (day 3–day 6) on the metabolic activity of the cells in comparison to the control was analyzed by means of one-way ANOVA with Dunnett’s multiple comparison test, using the AUCs from day 3 until day 6. Significant changes are indicated in the graphs. Underlying data are available at http://doi.org/10.5281/zenodo.1169306 (Clinical_chemistry_parameters).

The release of inflammatory factors was selectively affected by different APAP concentrations (Figure 4).

Release rates of the prostaglandin PGE2 (Figure 4A) showed a peak immediately after cell isolation followed by a rapid decline to basal levels. Upon APAP exposure, PGE2 release rates were characterized by a significant (*p* < 0.05) increase in bioreactors treated with 30 mM APAP as compared with the control group, indicating induction of PGE2 secretion at high APAP doses. In contrast, the cytokine IL-6 showed a general decrease in values in all groups during the culture course, and AUCs upon APAP incubation were similar in APAP-treated and untreated cultures (Figure 4B).

### 3.2. Gene Expression Analysis

**Figure 5:**
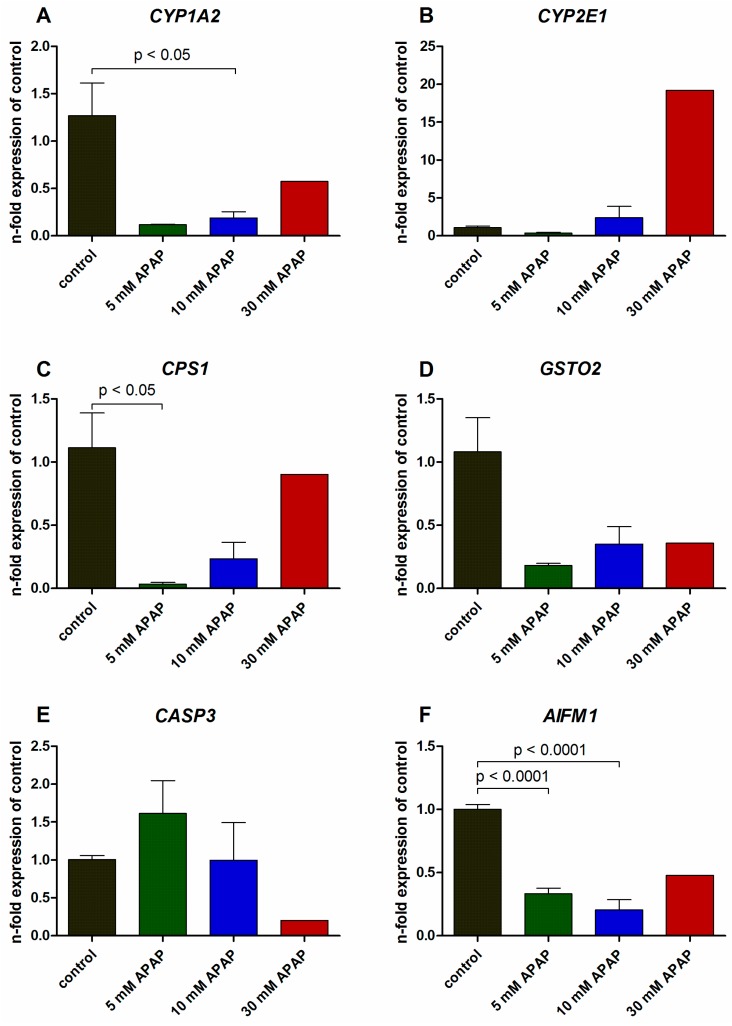
Gene expression analysis of primary human liver cells after culture in bioreactors treated with 5 mM, 10 mM or 30 mM acetaminophen (APAP) in comparison to untreated bioreactors used as control group. The figure shows the gene expression of cytochrome P450 family 1, subfamily A, polypeptide 2 (*CYP1A2*, (**A**)), cytochrome P450 family 2, subfamily E, polypeptide 1 (*CYP2E1*, (**B**)), carbamoyl phosphate synthetase I (*CPS1*, (**C**)), glutathione S-transferase omega 2 (*GSTO2*, (**D**)), caspase 3, apoptosis-related cysteine peptidase (*CASP3*, (**E**)) and apoptosis-inducing factor, mitochondria-associated, 1 (*AIFM1*, (**F**)). Expression data were normalized to the house-keeping gene glyceraldehyde-3-phosphate dehydrogenase and were calculated relative to the untreated control using the ΔΔCt-method. Data are shown as means ± SEM (control *n* = 4; 5 mM APAP *n* = 2; 10 mM APAP *n* = 3, 30 mM APAP *n* = 1). Differences between the control and 5 mM or 10 mM APAP were calculated using one-way ANOVA with Dunnett’s multiple comparison test and significant changes are indicated in the graphs. Underlying data are available at http://doi.org/10.5281/zenodo.1169306 (qRT_PCR).

The expression of genes involved in drug metabolism, antioxidant reactions, urea synthesis, and apoptosis was influenced individually by APAP exposure (Figure 5).

The genes encoding for *CYP1A2* (Figure 5A) and *CYP2E1* (Figure 5B) were strongly reduced in cultures exposed to 5 mM APAP as compared to the untreated control bioreactors, followed by a stepwise increase at higher APAP concentrations. While *CYP1A2* expression was lower in all APAP-treated groups than in the control, the expression of *CYP2E1* increased by 20-fold in the group treated with 30 mM APAP. The genes encoding for carbamoyl phosphate synthetase I (*CPS1*, Figure 5C) and glutathione S-transferase omega 2 (*GSTO2*, Figure 5D) showed a similar expression pattern, characterized by a 25-fold (*CPS1*, *p* < 0.05) resp. 4.5-fold (*GSTO2*) reduction in bioreactors treated with 5 mM APAP, and a successive increase at higher drug concentrations, without reaching the values of the control group. A different effect of APAP on gene expression was observed for the genes associated with apoptosis, namely caspase 3, apoptosis-related cysteine peptidase (*CASP3*, Figure 5E), and apoptosis-inducing factor, mitochondria-associated, 1 (*AIFM1*, Figure 5F). *CASP3* showed a slight increase in expression at 5 mM APAP, which was followed by a successive decline in the groups exposed to 10 or 30 mM APAP, whereas *AIFM1* expression was reduced in all APAP-treated groups, with significantly lower values in bioreactors treated with 5 or 10 mM APAP (*p* < 0.0001).

### 3.3. Histological and Immunohistochemical Analysis

**Figure 6:**
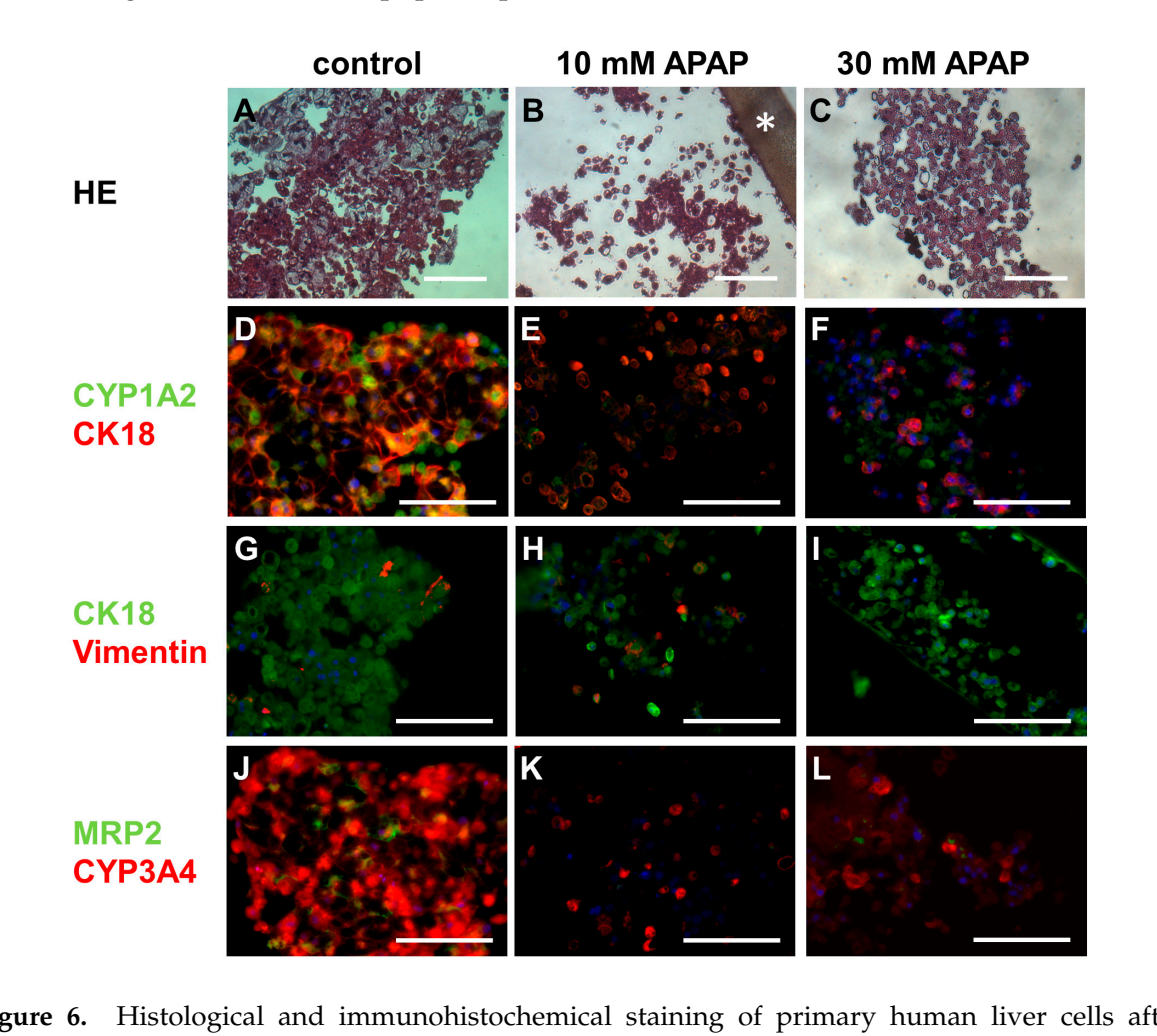
Histological and immunohistochemical staining of primary human liver cells after culture in untreated bioreactors (control) or those treated with 10 mM or 30 mM acetaminophen (APAP). The figure shows the hematoxylin-eosin stain (HE, (**A**–**C**)); double staining of cytochrome P450 family 1, subfamily A, polypeptide 2 (CYP1A2) and cytokeratin 18 (CK18) (**D**–**F**), CK18, and vimentin (**G**–**I**); and double staining of multidrug resistance-associated protein 2 (MRP2) and cytochrome P450 family 3, subfamily A, polypeptide 4 (CYP3A4) (**J**–**L**). The asterisk in (**B**) marks a hollow-fiber capillary membrane. Nuclei were counter-stained with Hoechst 33342 (blue). Scale bars correspond to 100 µm. Source pictures are available at http://doi.org/10.5281/zenodo.1169306 (Histology_Immnunofluorescence).

Histological investigation and immunofluorescence labeling of hepatic antigens in untreated cultures (control) or those exposed to 10 or 30 mM APAP showed a clear effect of APAP on the tissue organization and distribution pattern of cell-specific markers in bioreactor cultures (Figure 6).

As shown by HE staining (Figure 6A–C), primary human liver cells cultured in control bioreactors were associated in tissue-like cell aggregates between the hollow-fiber capillaries. Cell clusters contained primarily hepatocytes characterized by a large cytoplasm and a round or polygonal shape. The majority of cells appeared morphologically intact. Upon treatment with 10 or 30 mM APAP, cell aggregates and cell–cell connections were partly dissolved, resulting in the occurrence of numerous isolated cells. Most cells displayed a small and condensed cytoplasm and a lack of demarcation of cell nuclei, indicating necrotic and/or apoptotic processes.

Immunofluorescence staining confirmed the finding of cell damage and partial disintegration of cell aggregates upon APAP application. In control cultures, the hepatocyte-specific markers CK18 and CYP1A2 (Figure 6D–F) mostly showed an evenly distributed staining. The cytoskeletal marker CK18 was primarily expressed at cell margins, forming a network-like staining pattern, while CYP1A2 was mainly localized in the cytoplasm. In cultures treated with 10 or 30 mM APAP sparse and irregular staining of CK18 and CYP1A2 was observed. Staining of nuclei with Hoechst 33,342 revealed a condensed cytoplasm of most cells and furthermore, the occurrence of isolated nuclei devoid of cytoplasm, as an additional indication of cell death. Double-staining of CK18 and vimentin (Figure 6G–I) showed the presence of some non-parenchymal cells (vimentin-positive) between hepatocytes (CK18-positive), both in control bioreactors and those treated with 10 mM APAP. In contrast, no vimentin-positive cells were detected in cultures exposed to 30 mM APAP, indicating a loss of non-parenchymal cells. Expression of CYP3A4 (Figure 6J–L) was observed in the cytoplasm of most cells in control bioreactors and was still expressed in part of the cells after APAP treatment. The biliary transporter MRP2 being localized in plasma membranes of adjacent cells was detected in around 40–50% of the cells in control cultures. In bioreactors subjected to 10 or 30 mM APAP, the fraction of MRP2 positive cells was decreased to less than 10%.

## 4. Discussion

In order to precisely predict the hepatotoxicity of compounds during drug development, validated human in vitro models are necessary. Models based on a 3D environment have proven to more accurately reflect the human body compared to conventional 2D models [6]. Various microfluidic culture systems were developed to minimize the amounts of cells and culture materials [12,13]. The microscale 3D bioreactor used in this study is based on an existing four-compartment hollow-fiber technology [14,15,16,17] and was down-scaled to a cell compartment volume of 100 µL and a cultivated cell number of 10 million primary human liver cells.

To demonstrate the suitability of the microscale bioreactor system for hepatotoxicity studies, APAP was applied as a gold-standard test substance in concentrations of 5, 10, or 30 mM over a time period of three days. APAP toxicity manifests in many different ways as reviewed by Hinson et al. (2010), such as glutathione depletion, formation of toxic protein adducts, enzyme and cytokine release, and histological alterations [22]. In this study, the focus was on clinical chemistry parameters allowing regular evaluation of APAP toxicity during culture, followed by end-point analyses allowing the judgement of alterations in tissue integrity as well as protein and gene expression.

The time course of clinical chemistry parameters measured in the culture perfusate was generally characterized by an initial peak on the first day of culture. This increase can be ascribed to the cell isolation process, which leads to high levels of cell stress causing the release of intracellular enzymes and metabolites. In addition, disruption of cell–cell and cell–matrix contacts and consequently the loss of cell polarization were described [23]. Liver cells may also become pre-activated due to reperfusion injury associated with oxidative stress [24]. Both disruption of tissue integrity and activation of inflammatory signaling, are associated with dedifferentiation processes starting already during isolation [5].

In concordance with other studies [7,25] we observed dose-dependent toxic effects of APAP on primary human liver cells cultured in the device. Glucose and lactate production rates measured as parameters for energy metabolism showed a dose-dependent decrease from day 3 (beginning of APAP dosing) onwards indicating an impaired cell viability and functionality. The suitability of glucose consumption and lactate production to detect drug-induced changes in cell viability of hepatic cell cultures was previously shown [26]. In contrast to assays based on substrate conversion, for example, MTT or cell titer blue assay, measurement of glucose and lactate allows a regular monitoring of cell activity over time without intervening into the cell metabolism.

Additionally, the enzyme release was measured as an indicator for necrosis or for secondary necrosis following apoptosis [27,28]. Serum levels of the cytoplasmic enzyme LDH are routinely measured in clinical settings in order to assess cell damage within pathological processes, including liver injury [29,30]. AST is distributed both in the cytoplasm and mitochondria of hepatocytes [31]; mild cell injury causes the release of cytosolic enzymes, whereas severe liver damage leads to the release of both, cytoplasmic and mitochondrial enzymes. All experimental groups showed an initial peak of enzyme values on day 1 in consistence with other studies [32]. The absence of an increase in enzyme release during the APAP treatment period is in accordance with previous results investigating diclofenac toxicity in 3D bioreactors [15]. Similarly, periodic treatment of hepatocyte cultures with 18.6 mM APAP for 20 days had no significant effect on LDH release [33]. The absence of response in hepatic enzyme release may be explained by the exhaustion of cytosolic enzyme stores in the initial culture phase, which can be ascribed to cell stress during cell isolation. In a study investigating liver enzyme concentrations in the cytosol and in the supernatant of primary human hepatocytes exposed to APAP, the amount of secreted enzyme relative to the total enzyme content showed a significant increase upon drug application [25]. Hence, the determination of both extracellular and intracellular LDH activity could provide more conclusive results on the actual effect of APAP on enzyme release. However, this would require daily lysis of cell samples, which is difficult to realize in complex 3D culture systems.

Ammonia, a product of amino acid metabolism, is toxic in high concentrations and is therefore converted to urea by hepatocytes. Hence, through the analysis of urea synthesis and ammonia depletion, conclusions can be drawn regarding the functionality of cultured hepatocytes [34]. The observed initial increase in urea and ammonia production rates can be related to cell stress due to cell isolation, in accordance with the observed peaks in enzyme release. The finding of significantly increased ammonia release rates after dosing with 10 or 30 mM APAP from day 3 onwards indicate a dose-dependent influence of APAP on the nitrogen metabolism of the cells. This finding is supported by the fact that APAP toxicity causes mitochondrial dysfunction [25], since part of the enzymes involved in the urea cycle are located in the mitochondria. Moreover, these observations emphasize the suitability of ammonia as a sensitive parameter for hepatocyte functionality and hepatic drug toxicity.

The inflammatory factors PGE2 and IL-6 showed a different course upon drug application. A dose-dependent increase from day 3 (beginning of APAP dosing) onwards was detected for PGE2, a pro-inflammatory factor with immunosuppressive activity, indicating an accumulation of APAP-induced inflammation. Since PGE2 is typically produced by cell types such as endothelial cells and cells of the immune system [35], the finding of an increased PGE2 release confirms the presence of non-parenchymal cells in the liver bioreactor. In contrast, the pro-inflammatory cytokine IL-6, which is secreted by different liver cell types, including hepatocytes [36], showed no significant response to APAP exposure. PGE2 inhibits the production of IL-6 as a mechanism of limiting excessive immune reactions [37], which may be the reason for the lack of IL-6 response to APAP treatment.

Since production rates of metabolic parameters were normalized to the initial cell number, the results from the medium analysis represent the combined effect of cell number and functionality. Thus, values do not allow a distinction between a change either of cell number or of cell activity in the applied culture system.

In the liver, APAP is metabolized mainly by *CYP2E1* to the toxic N-acetyl-*p*-benzochinonimin (NAPQI) [38,39]. Gene expression analysis of the cells subsequent to APAP exposure in comparison to untreated control bioreactors revealed a dose-dependent increase in *CYP2E1* expression especially for 30 mM APAP. This result indicates that high concentrations of APAP lead to an immediate upregulation of *CYP2E1*, which can be seen as a mechanism to accelerate APAP metabolism. In addition, we observed a decrease in gene expression of *CYP1A2* upon APAP treatment compared to the control bioreactor, though less pronounced at increasing APAP concentrations. The contribution of CYP1A2 to APAP metabolism is controversially discussed. While studies considering rat and human liver microsomes [40,41] or recombinant human CYP P450 enzymes [42] showed that CYP1A2 is involved in the metabolism of APAP, other publications, performed in adult human volunteers, reported no direct association of CYP1A2 with APAP depletion [38,43]. These contradictory findings may be caused by different conditions in in vitro experiments as compared with the in vivo situation.

The glutathione S-transferases (GSTs) are enzymes catalyzing the neutralization of free radicals and active drug components using glutathione as reducing agent, which is the main step in phase 2 detoxification [44]. In APAP metabolism, GSTs catalyze the formation of a NAPQI-glutathione adduct, which is then primarily secreted into bile by passing the apical membrane transporter protein MRP2 [45]. Treatment of hepatocytes with APAP resulted in a decrease of *GSTO2* gene expression as compared to the control. Similar findings were reported by Wang et al. (2017), who observed a reduction of GST activities in a mouse model of APAP-induced liver injury [46]. The observed decrease in gene expression of *CPS-1*, an enzyme involved in the production of urea, is in accordance to our observations of increased ammonia release rates in the bioreactors treated with APAP, and further supports the assumption that APAP effects the nitrogen metabolism.

In contrast, the apoptosis-associated gene *CASP3* showed an increase in expression for the bioreactor treated with 5 mM APAP in comparison to the untreated controls, while no change was observed at 10 mM APAP and a decrease was detected after exposure to 30 mM APAP. This observation might be explained by a shift to necrotic cell death, in accordance with findings by Au and colleagues, who observed a transition from apoptosis to necrosis between 10 and 20 mM APAP exposure using HepG2 cells [47]. However, another apoptosis-associated factor, *AIFM1*, revealed reduced expression values for all drug-treated bioreactors as compared to the control. The pro-apoptotic function of AIFM1 is based on the activation of a caspase-independent pathway upon apoptotic stimuli, whereas its anti-apoptotic function is part of the regular mitochondria metabolism via NADH oxidoreduction [48]. As APAP treatment results in mitochondrial dysfunction, it might consequently also lead to a reduced gene expression of *AIFM1*. Since APAP is known to induce both necrosis and apoptosis [49], a more detailed characterization of the type of cell death would be of interest in future studies to differentiate between necrosis and apoptosis.

Histological and immunohistochemical analyses revealed that the cells cultured in control bioreactors were reorganized in tissue-like formations, which resembled those observed in larger scale bioreactors with higher initial cell amounts [14,16]. Typical structural and functional markers of hepatocytes, including CK18, CYP1A1 and CYP3A4 were regularly detected and showed an in vivo-like distribution pattern. Staining of MRP2, which is the main transporter for biliary excretion of acetaminophen sulfate [50,51], indicates cell polarization with formation of bile canaliculi. The characterization of the cells by means of cell-specific markers showed that in addition to hepatocytes, the aggregates also comprised non-parenchymal cells identified by vimentin staining. Since the non-parenchymal cells of the liver play a major role in drug-induced liver injury [52], the presence of these cells in liver models is critical to assess complex drug effects mediated by different liver cell populations.

Exposure to APAP at a concentration of 10 or 30 mM resulted in partial disintegration of cell aggregates and loss of cell integrity. In particular, MRP2 was rarely detectable in APAP-treated bioreactors indicating a depolarization of hepatocytes. This is in line with findings by Bhise and colleagues, who showed a massive reduction of MRP2 immunostaining after treatment of hepatic spheroids composed of hepatoma cells with 15 mM APAP [53]. The reduction of membrane transporters may lead to impaired excretion of APAP metabolites and therefore cause accumulation of toxic products in the cells, if glutathione is not sufficiently available.

In summary, we were able to detect dose-dependent hepatotoxic effects of 5 to 30 mM APAP on primary human hepatocytes cultured in the microscale 3D bioreactor. Au and colleagues identified hepatotoxic influences of APAP at 10 mM using spheroids comprising HepG2 cells and fibroblasts cultured on a microfluidic platform [47]. Other microfluidic studies using HepG2 cells found a reduction of cell viability by more than 50% upon treatment with 15 mM APAP [53] resp. 20 mM APAP [54]. However, in the human body, plasma concentrations of 0.5 to 3 mM APAP were observed in overdose scenarios [55]. A potential reason for the discrepancy between toxic APAP concentrations in vivo and in vitro can be seen in the contribution of systemic influences, such as nutritional status [56] and blood cells [57], to APAP toxicity. In addition, the non-parenchymal liver cells, such as sinusoidal endothelial cells [58] and Kupffer cells [59], have been shown to play a role in APAP toxicity. Although in the present study the obtained mixture of primary liver cells after enzymatic digestion of the organ was used without further purification of hepatocytes, the number of non-parenchymal cells might not have been sufficient to correctly imitate the human in vivo liver.

Hence, future studies would be of interest to further investigate the role of individual liver cell populations in APAP metabolism and toxicity. The addition of non-parenchymal liver cells to the microscale 3D liver culture in ratios comparable to the in vivo situation could increase its sensitivity for APAP toxicity. Methods for isolation of endothelial cells, Kupffer cells and stellate cells from human liver tissue were recently described [18] and can be used to provide defined amounts of these cells for human liver cell models. Other microfluidic systems attempt to recapitulate the microarchitecture of the liver sinusoid by providing several compartments for the different liver cell types [60,61]. A further important factor in drug susceptibility might be the oxygen concentration, as indicated by results from co-cultures of primary rat hepatocytes and fibroblasts, which proved to be more sensitive to APAP exposure in low-oxygen regions [62]. Thus, the creation of physiological oxygen gradients might increase the predictive power of in vitro drug effects. To assess systemic effects mediated by other organs, integration of the microscale 3D liver bioreactor into a multi-organ platform would be an attractive approach. In this context, the structure of the bioreactor provides suitable conditions for realization of microscale systems integrating various organ constructs, such as kidney, heart, and lung.

A general limitation of microscale systems can be seen in the small amount of cell material available for end-point analyses such as immunohistochemistry, qPCR, or Western blots. Thus, studies investigating microscale liver tissues often show results from only one end-point analysis, mostly immunohistochemistry or Western blotting [32,47,53,54,62]. Hence, the implementation of analytic methods allowing analyses from minimal cell numbers is required to generate a larger range of data from microscale systems.

## 5. Conclusions

In conclusion, the results from APAP application in this study demonstrate that the microscale 3D liver bioreactor provides a useful tool to detect hepatotoxic effects of drugs in a perfused human in vitro culture environment. Our observations emphasize the potential of clinical chemistry parameters, such as lactate production and ammonia release, as sensitive parameters for monitoring dose-dependent hepatotoxic effects throughout the culture period. The analysis of inflammatory factors showed that mainly PGE2 was affected by APAP exposure in the model. The toxic effect of APAP in the in vitro model was confirmed by end-point analyses, including histological and immunohistochemical evaluation and PCR analysis. In order to increase the sensitivity of the present microscale 3D liver bioreactor for toxicity studies at physiologically relevant drug concentrations, co-cultures supplemented with different non-parenchymal cell types at physiological ratios, and also creation of defined oxygen gradients could be applied in future studies.

## Figures and Tables

**Table 1:** Bioreactor perfusion parameters and operation conditions.

Parameter	Set Values during Operation
Recirculation rate	1 mL/min
Feed rate	0.6 mL/h (0–24 h)
	0.2 mL/h (from 24 h on)
Gas flow rate	4 mL/min
Concentration of CO_2_ in supplied gas mixture	3–6% ^1^
Temperature in bioreactor chamber	38 °C
pH value	7.35–7.45

^1^ The CO_2_ concentration was adjusted on demand to maintain a constant pH value in the system.

**Table 2:** Sample volumes and variation coefficients of analyzed clinical chemistry parameters.

Parameter	Required Volume	Variation Coefficient
Glucose	100 µL in total	4%
Lactate		3%
LDH	250 µL in total	1%
AST		4%
ALT		2.9%
GLDH		0.8%
Urea		1%
Ammonia	250 µL	2.2%
PGE2	200 µL	n.a.
IL-6	200 µL	n.a.

**Table 3:** Applied Biosystems TaqMan Gene Expression Assays^®^.

Gene Symbol	Gene Name	Assay ID
*AIFM1*	Apoptosis-inducing factor, mitochondria-associated, 1	Hs00377585_m1
*CASP3*	Caspase 3, apoptosis-related cysteine peptidase	Hs00234387_m1
*CPS1*	Carbamoyl-phosphate synthase 1, mitochondrial	Hs00157048_m1
*CYP1A2*	Cytochrome P450, family 1, subfamily A, polypeptide 2	Hs00167927_m1
*CYP2E1*	Cytochrome P450, family 2, subfamily E, polypeptide 1	Hs00559368_m1
*GAPDH*	Glyceraldehyde-3-phosphate dehydrogenase	Hs03929097_g1
*GSTO2*	Glutathione S-transferase omega 2	Hs01598184_m1

## Supplementary Materials

Raw data underlying the presented figures are available online on the data repository “Zenodo” under http://doi.org/10.5281/zenodo.1169306 with the doi:10.5281/zenodo.1169306.

Supplementary File 1
