# Supplementary figures and images for: Microscale 3D Liver Bioreactor for In Vitro Hepatotoxicity Testing under Perfusion Conditions
Source: Bioengineering (Basel). 2018 Mar 15;5(1):24. doi: 10.3390/bioengineering5010024 (PMC5874890; doi:10.3390/bioengineering5010024)

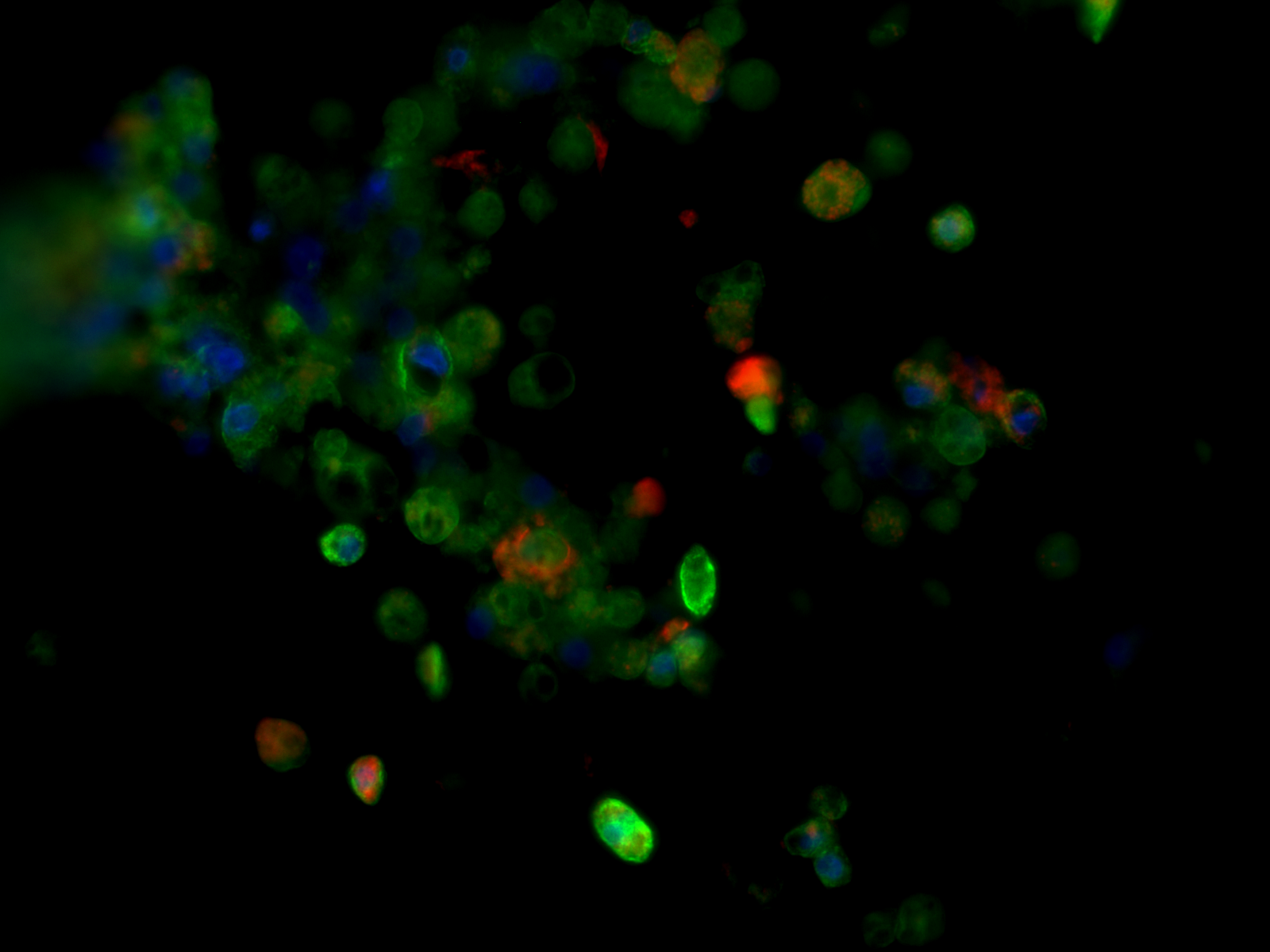

Supplement: Supplementary File 1 [file bioengineering-05-00024-s001.zip › Histology_Immnunofluorescence/CK18_Vim/10mM_CK18_Vim_40x_Composite.tif]

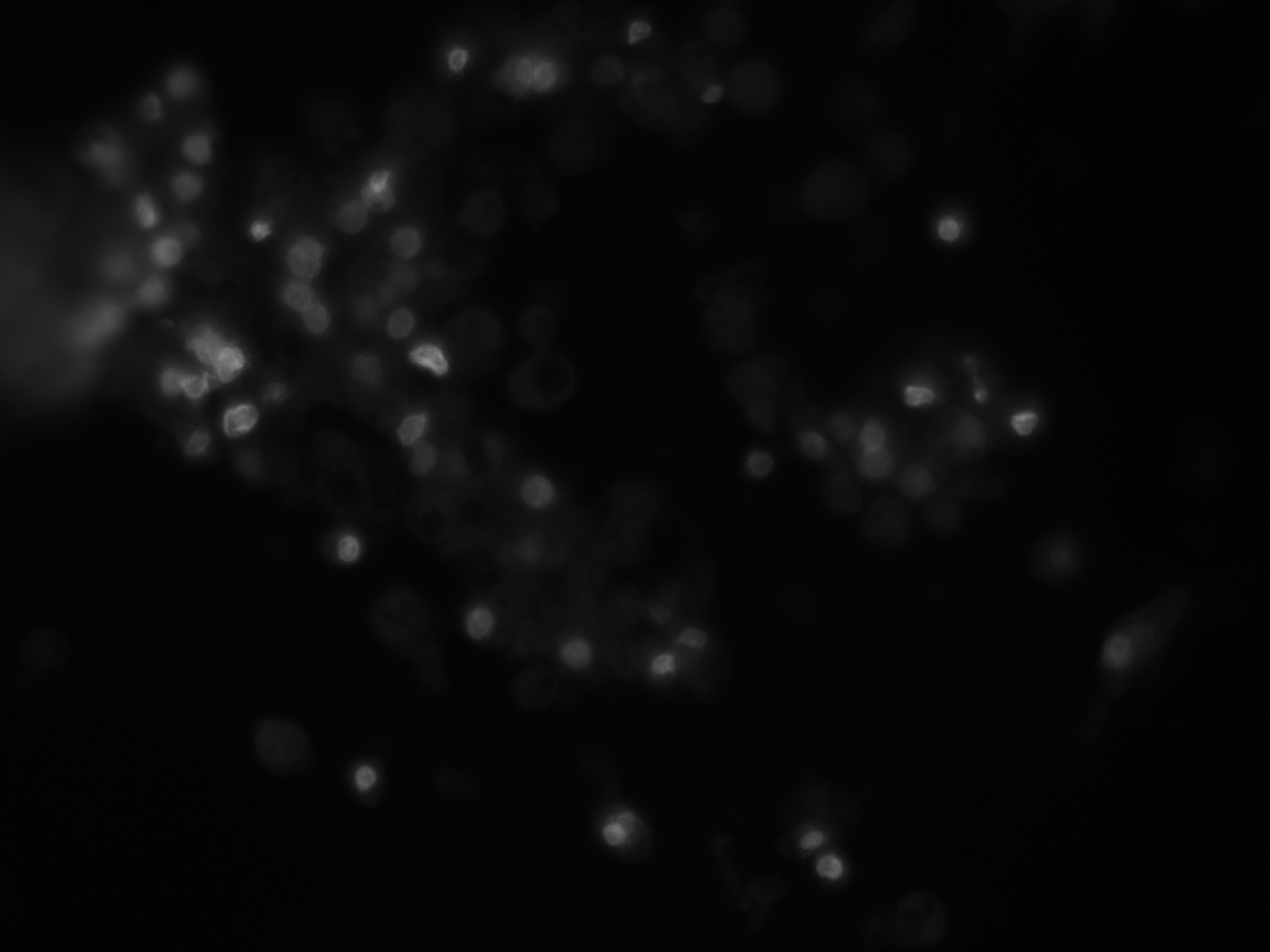

Supplement: Supplementary File 1 [file bioengineering-05-00024-s001.zip › Histology_Immnunofluorescence/CK18_Vim/10mM_CK18_Vim_40x_Dapi.tif]

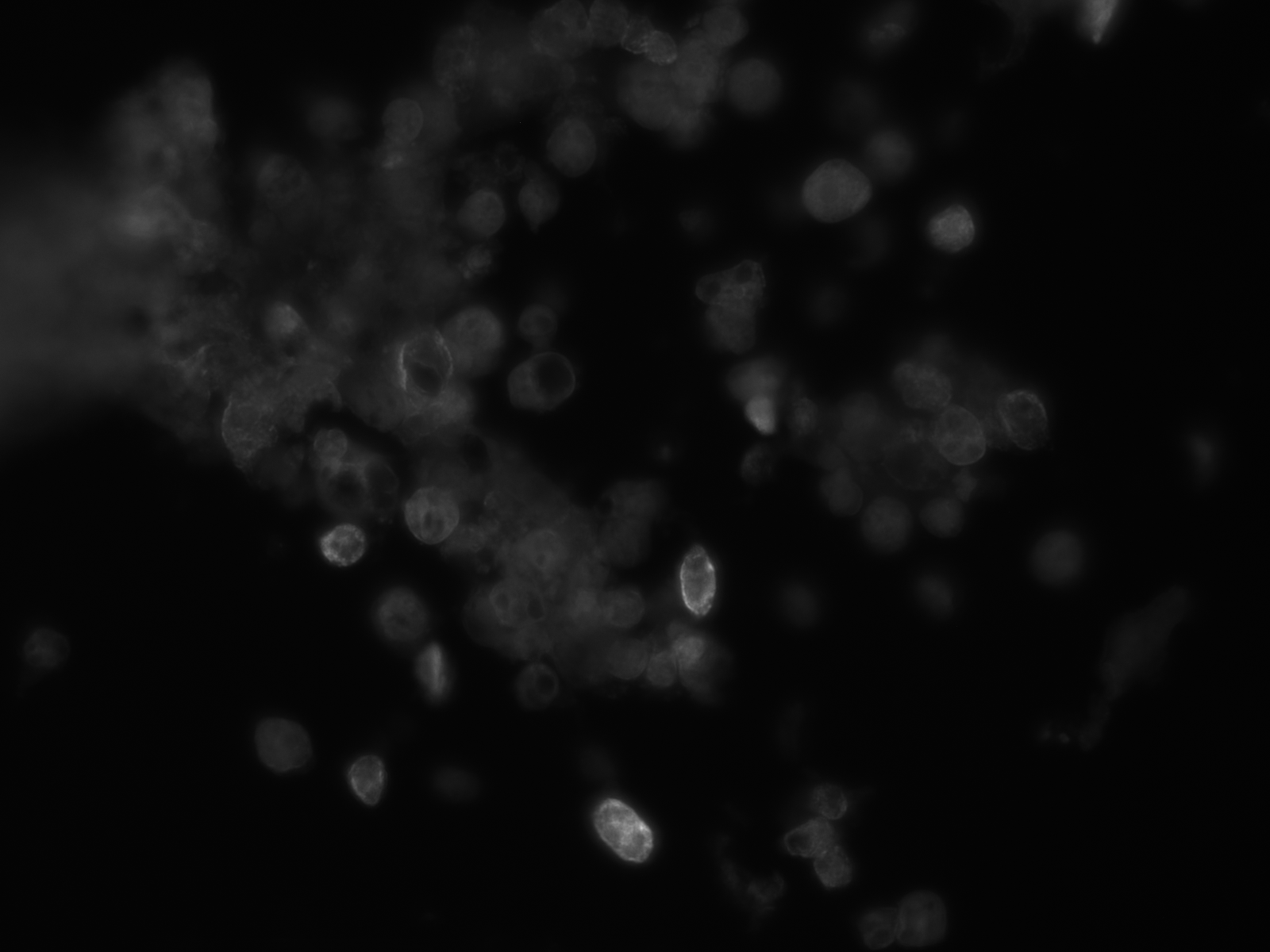

Supplement: Supplementary File 1 [file bioengineering-05-00024-s001.zip › Histology_Immnunofluorescence/CK18_Vim/10mM_CK18_Vim_40x_Green.tif]

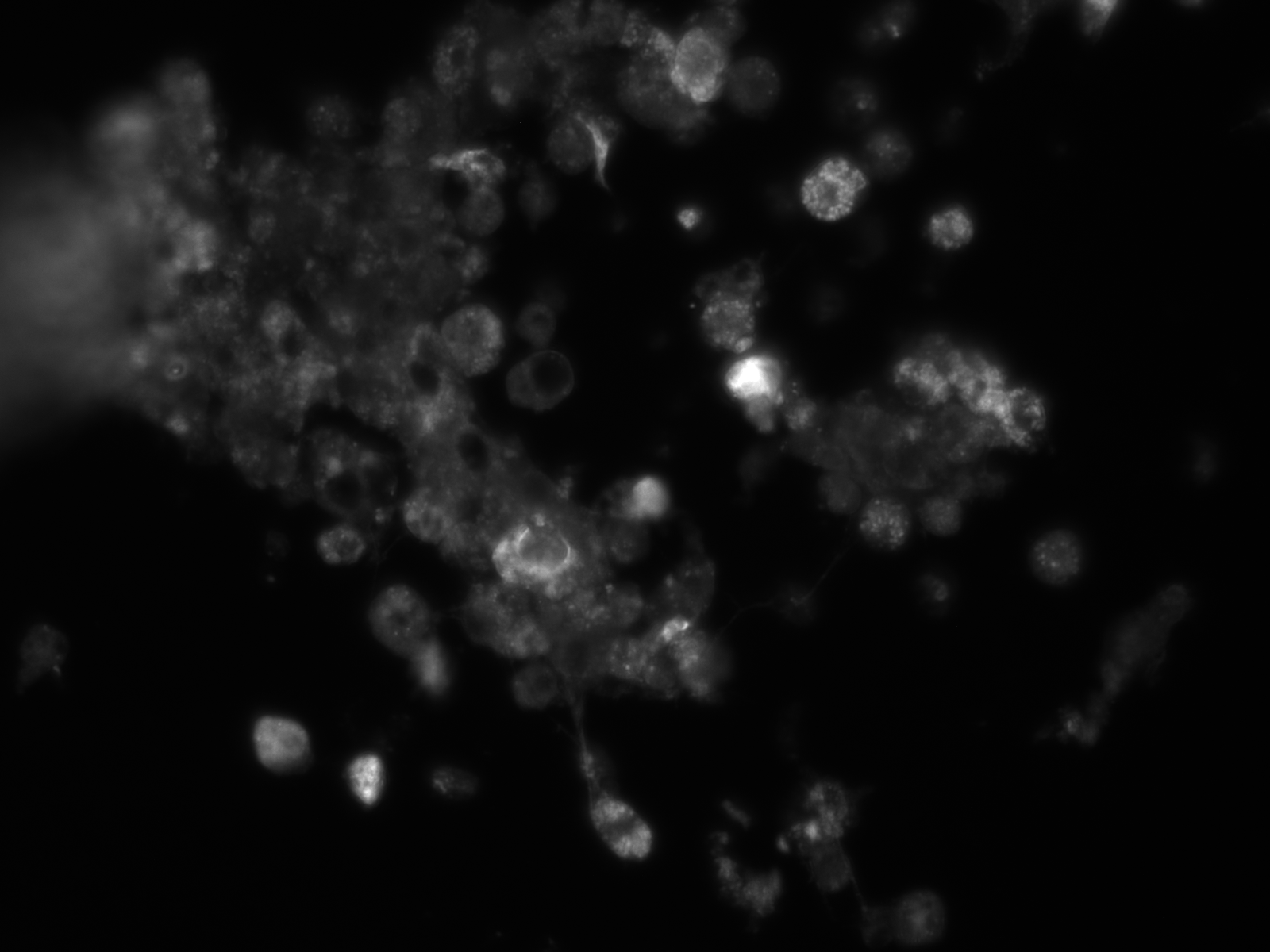

Supplement: Supplementary File 1 [file bioengineering-05-00024-s001.zip › Histology_Immnunofluorescence/CK18_Vim/10mM_CK18_Vim_40x_Red.tif]

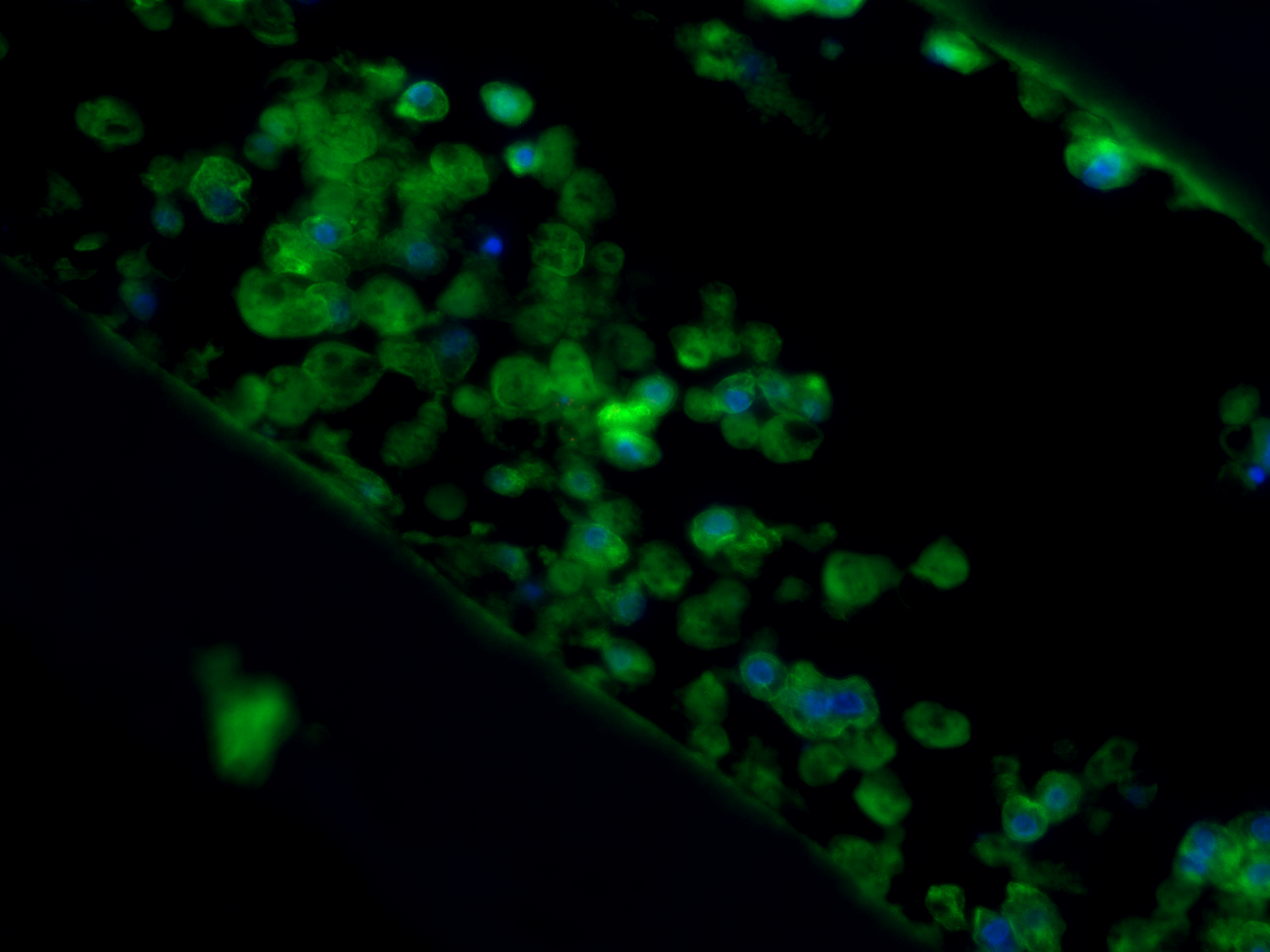

Supplement: Supplementary File 1 [file bioengineering-05-00024-s001.zip › Histology_Immnunofluorescence/CK18_Vim/30mM_CK18_Vim_40x_Composite.tif]

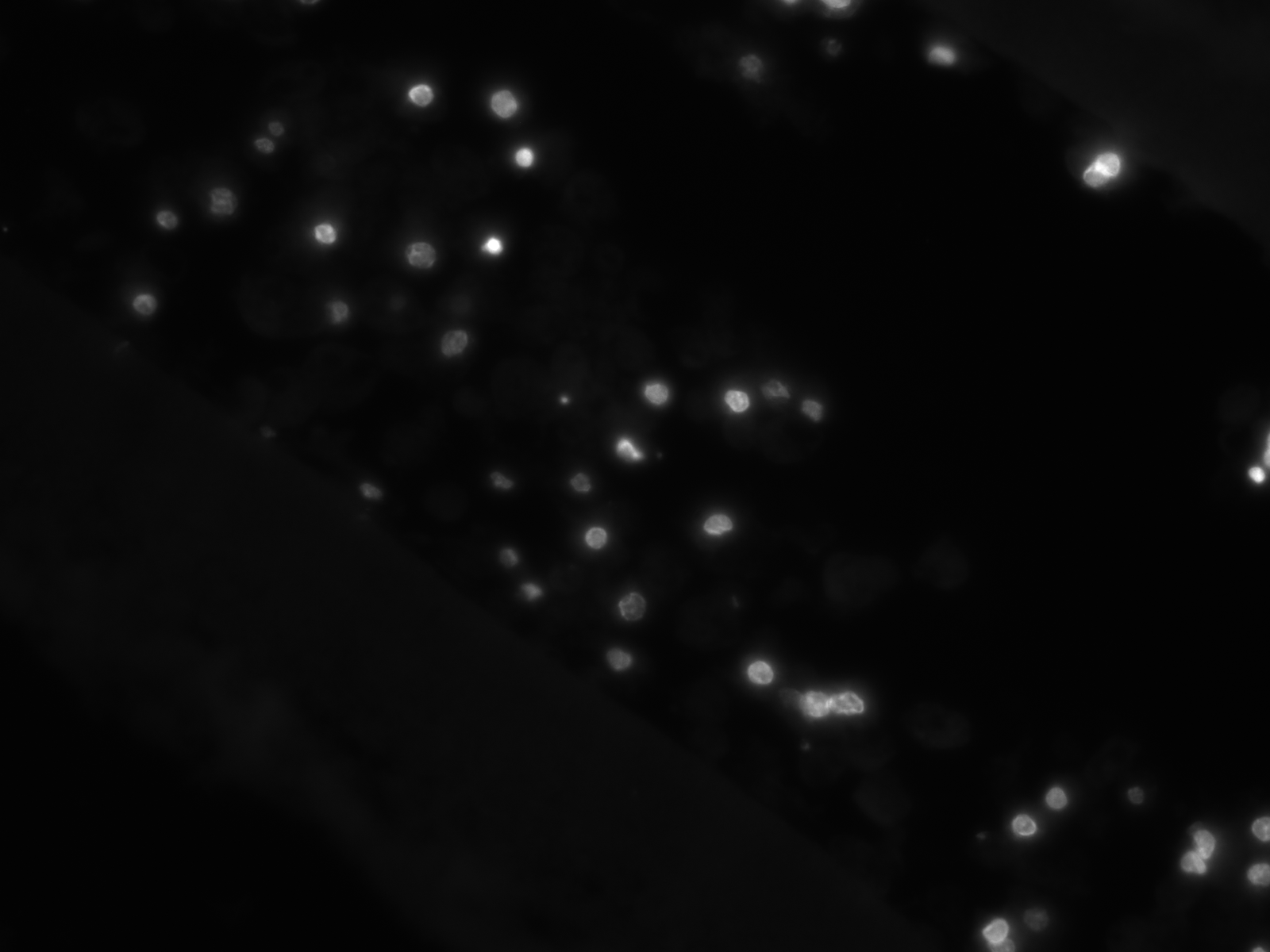

Supplement: Supplementary File 1 [file bioengineering-05-00024-s001.zip › Histology_Immnunofluorescence/CK18_Vim/30mM_CK18_Vim_40x_Dapi.tif]

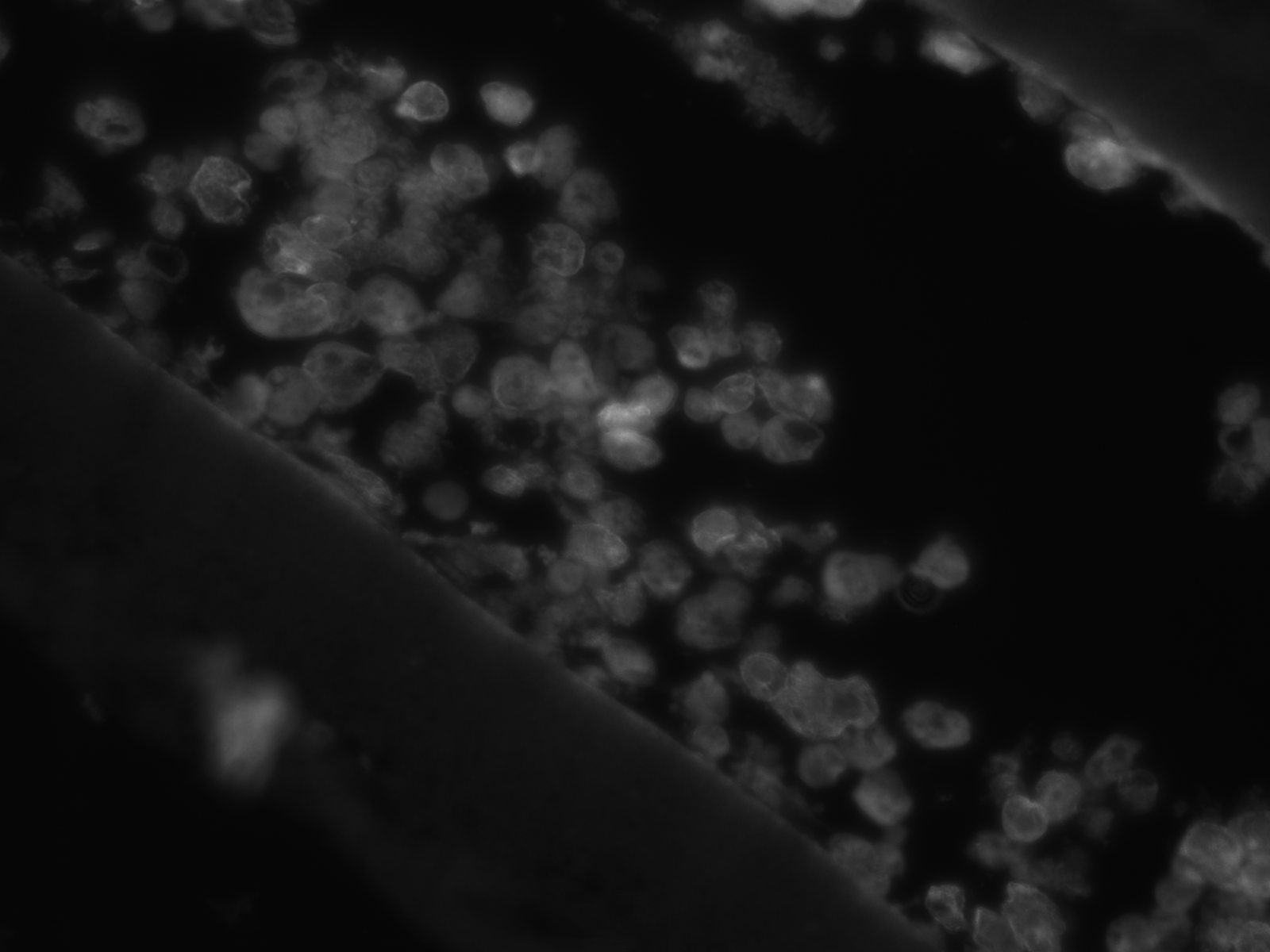

Supplement: Supplementary File 1 [file bioengineering-05-00024-s001.zip › Histology_Immnunofluorescence/CK18_Vim/30mM_CK18_Vim_40x_Green.tif]

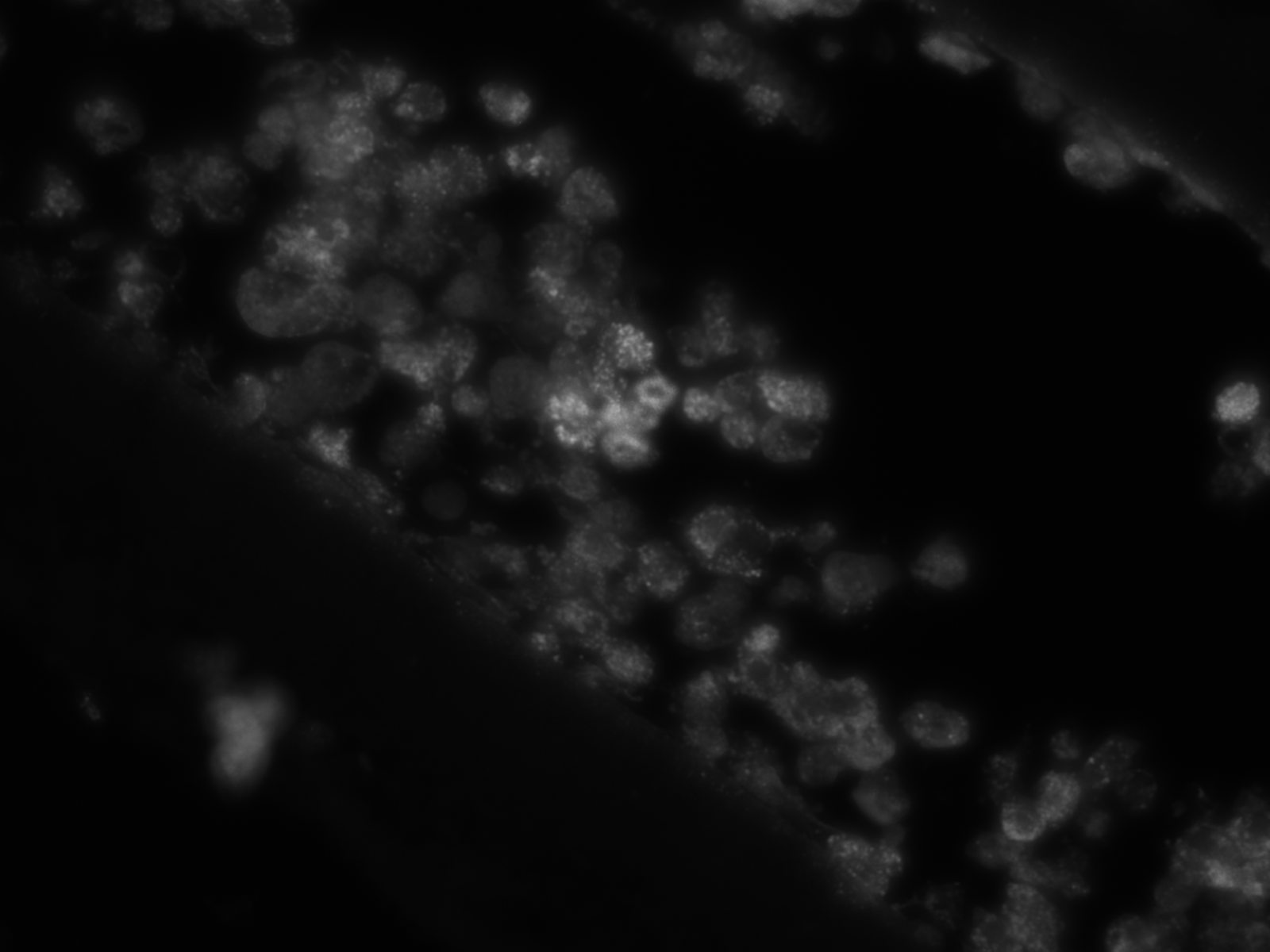

Supplement: Supplementary File 1 [file bioengineering-05-00024-s001.zip › Histology_Immnunofluorescence/CK18_Vim/30mM_CK18_Vim_40x_Red.tif]

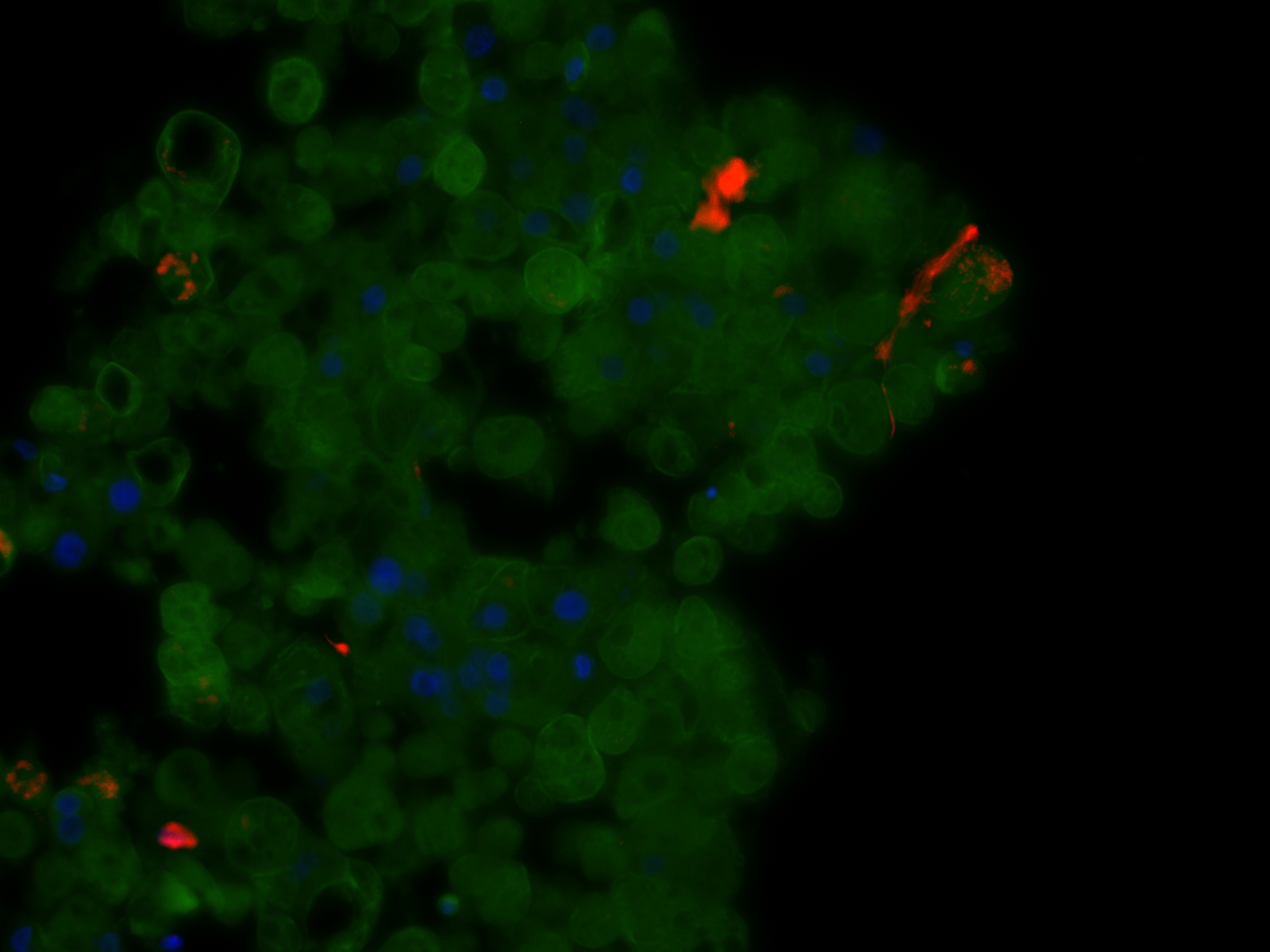

Supplement: Supplementary File 1 [file bioengineering-05-00024-s001.zip › Histology_Immnunofluorescence/CK18_Vim/Control_CK18_Vim_40x_Composite.tif]

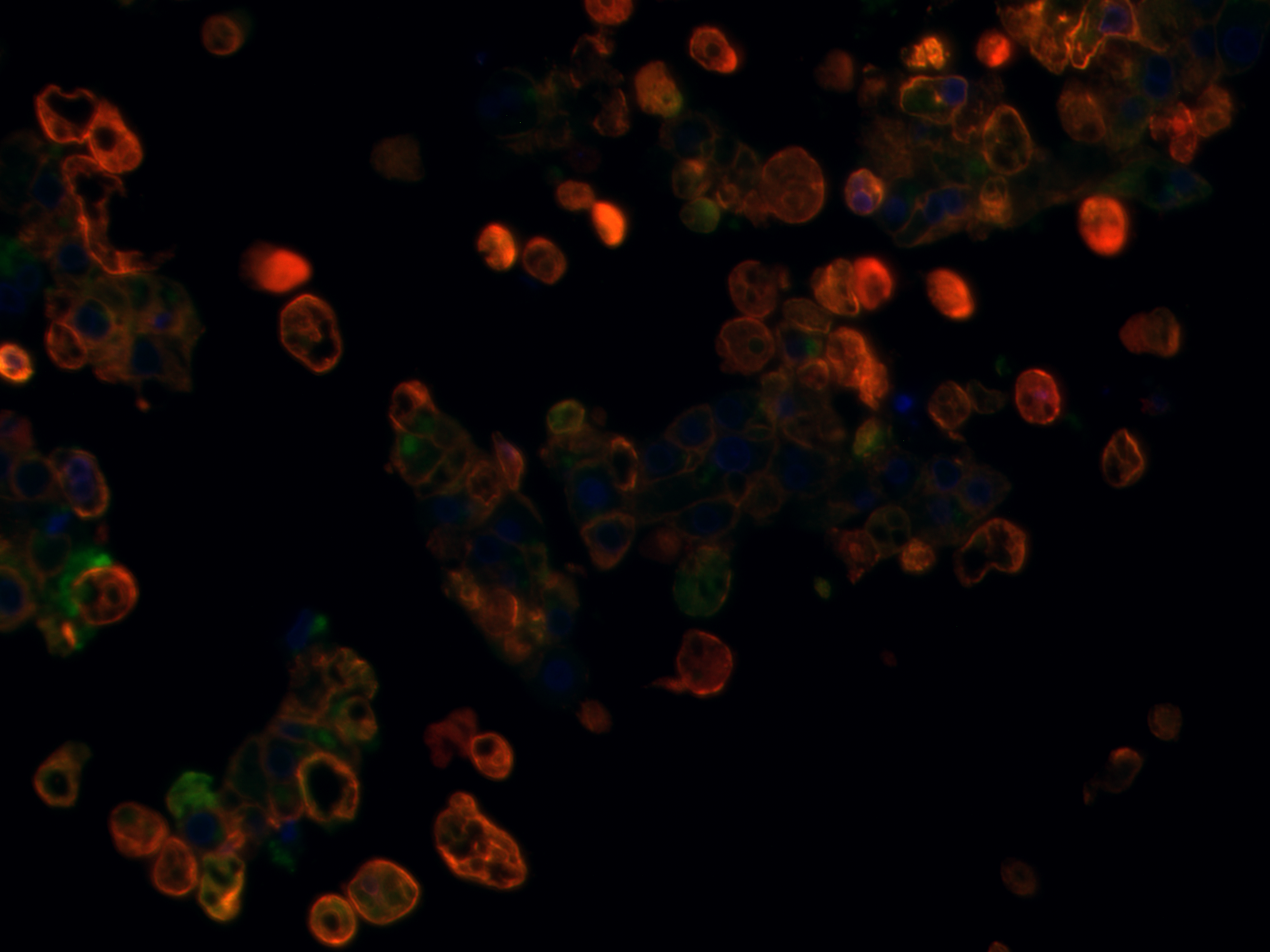

Supplement: Supplementary File 1 [file bioengineering-05-00024-s001.zip › Histology_Immnunofluorescence/CYP1A2_CK18/10mM_CYP1A2_CK18_40x_Composite.tif]

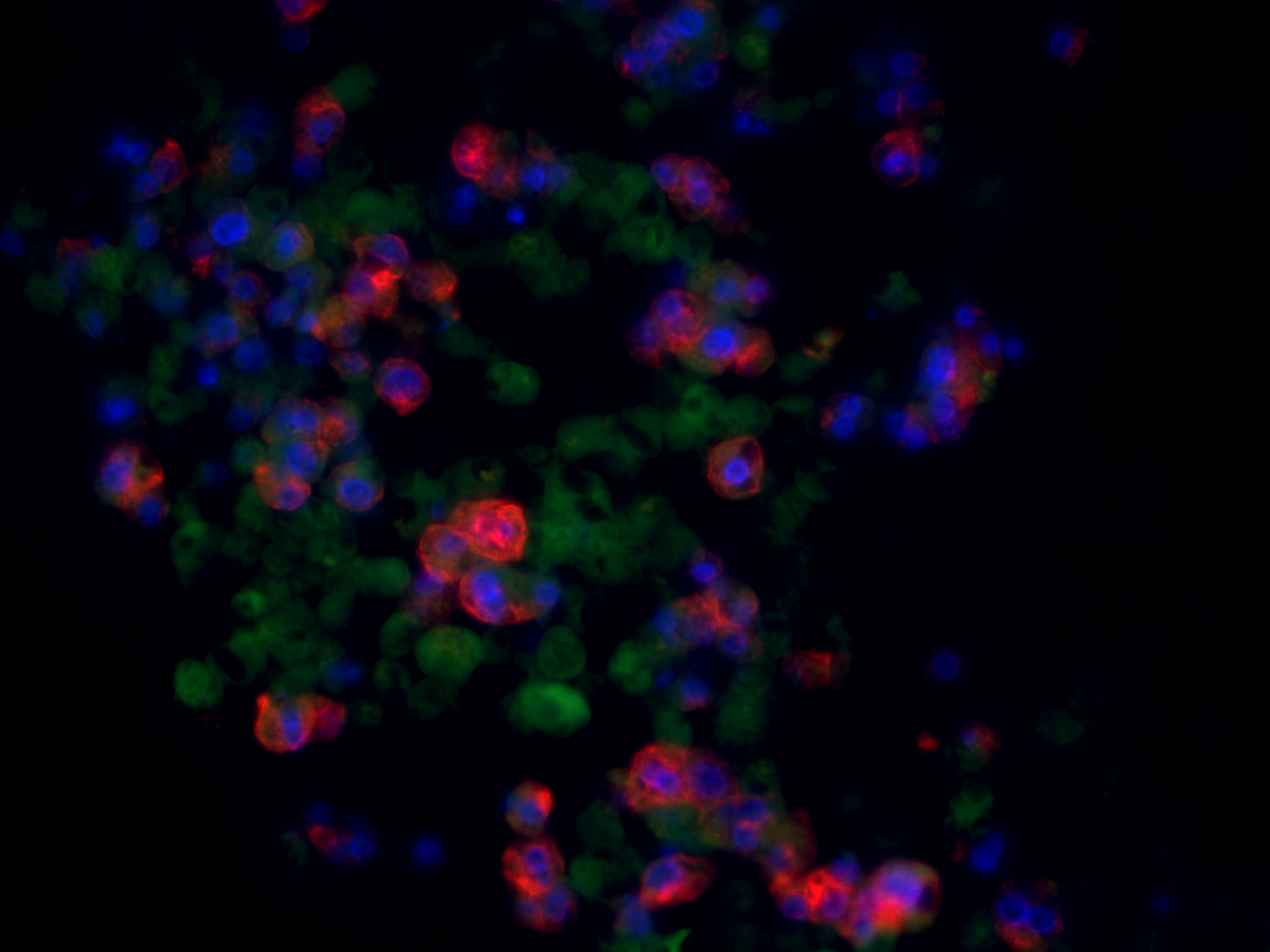

Supplement: Supplementary File 1 [file bioengineering-05-00024-s001.zip › Histology_Immnunofluorescence/CYP1A2_CK18/30mM_CYP1A2_CK18_40x_Composite.tif]

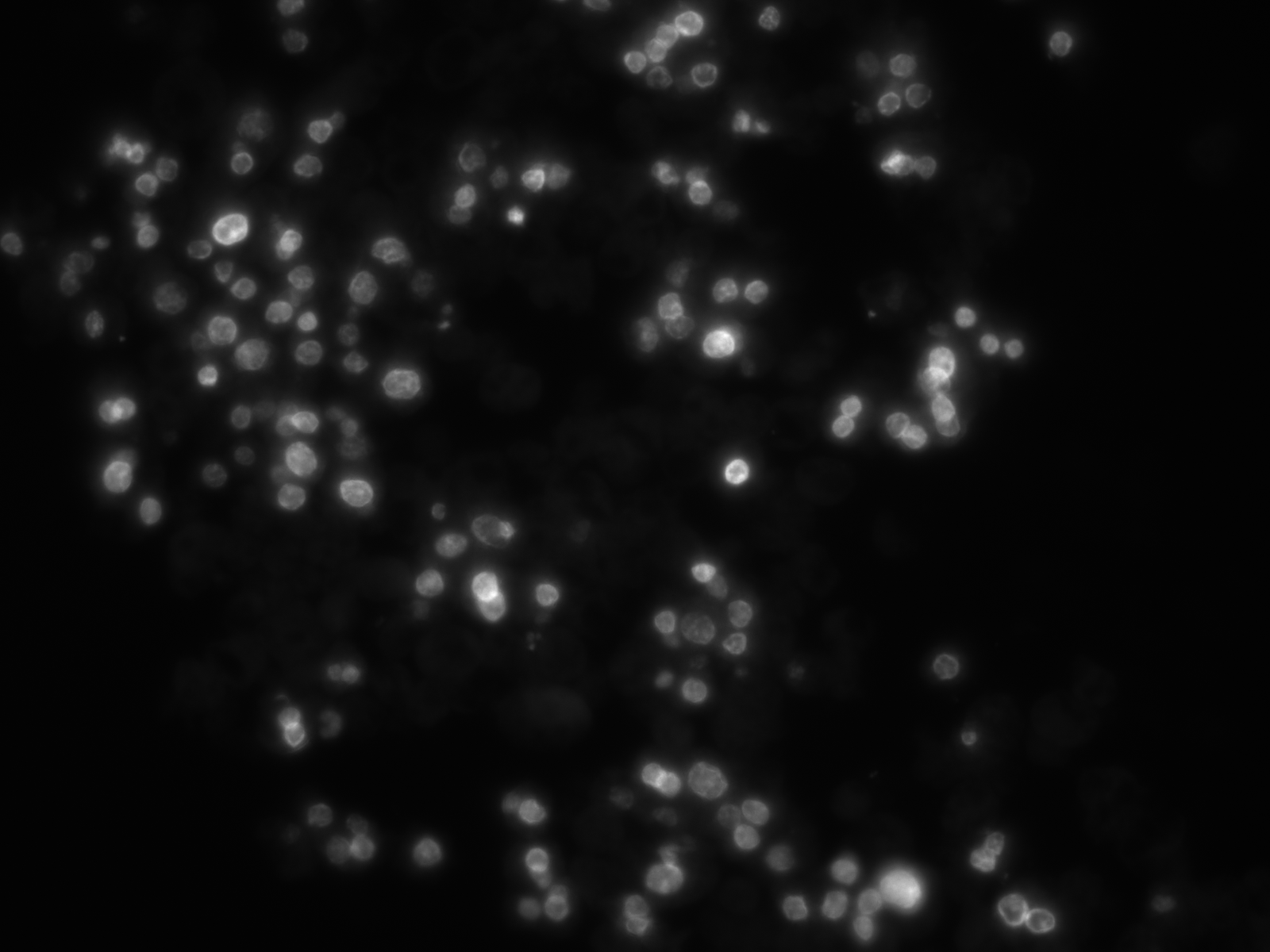

Supplement: Supplementary File 1 [file bioengineering-05-00024-s001.zip › Histology_Immnunofluorescence/CYP1A2_CK18/30mM_CYP1A2_CK18_40x_Dapi.tif]

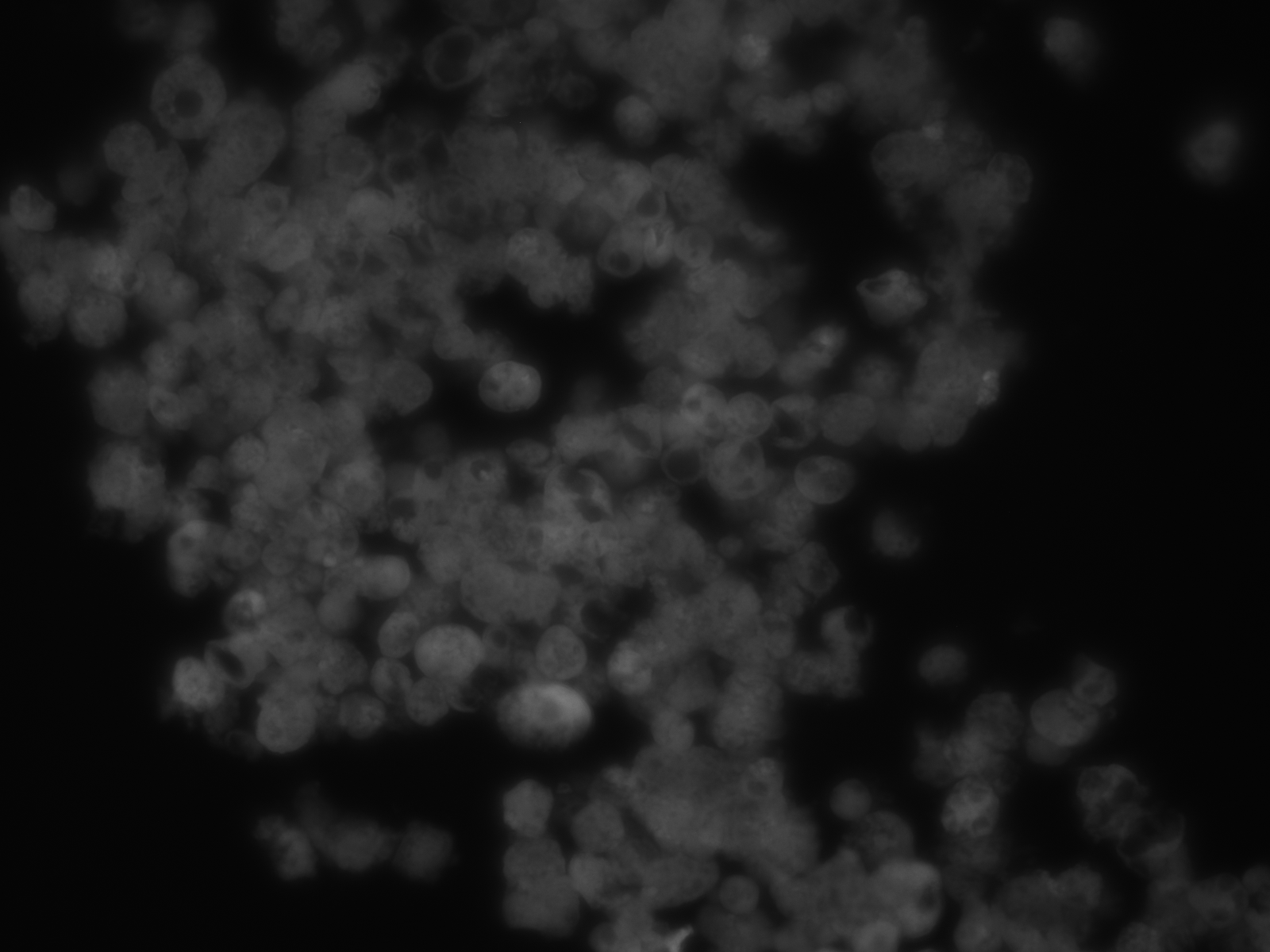

Supplement: Supplementary File 1 [file bioengineering-05-00024-s001.zip › Histology_Immnunofluorescence/CYP1A2_CK18/30mM_CYP1A2_CK18_40x_Green.tif]

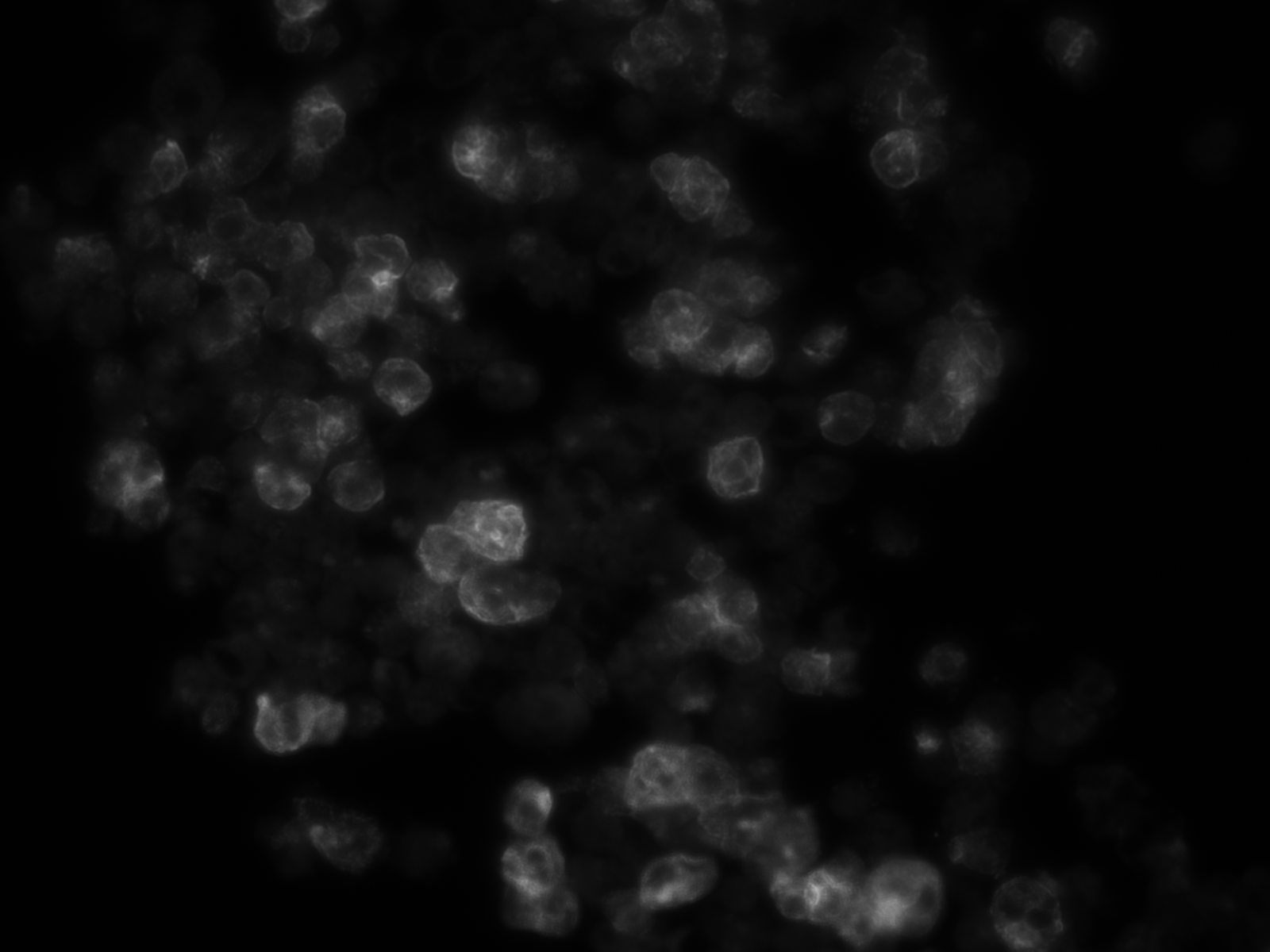

Supplement: Supplementary File 1 [file bioengineering-05-00024-s001.zip › Histology_Immnunofluorescence/CYP1A2_CK18/30mM_CYP1A2_CK18_40x_Red.tif]

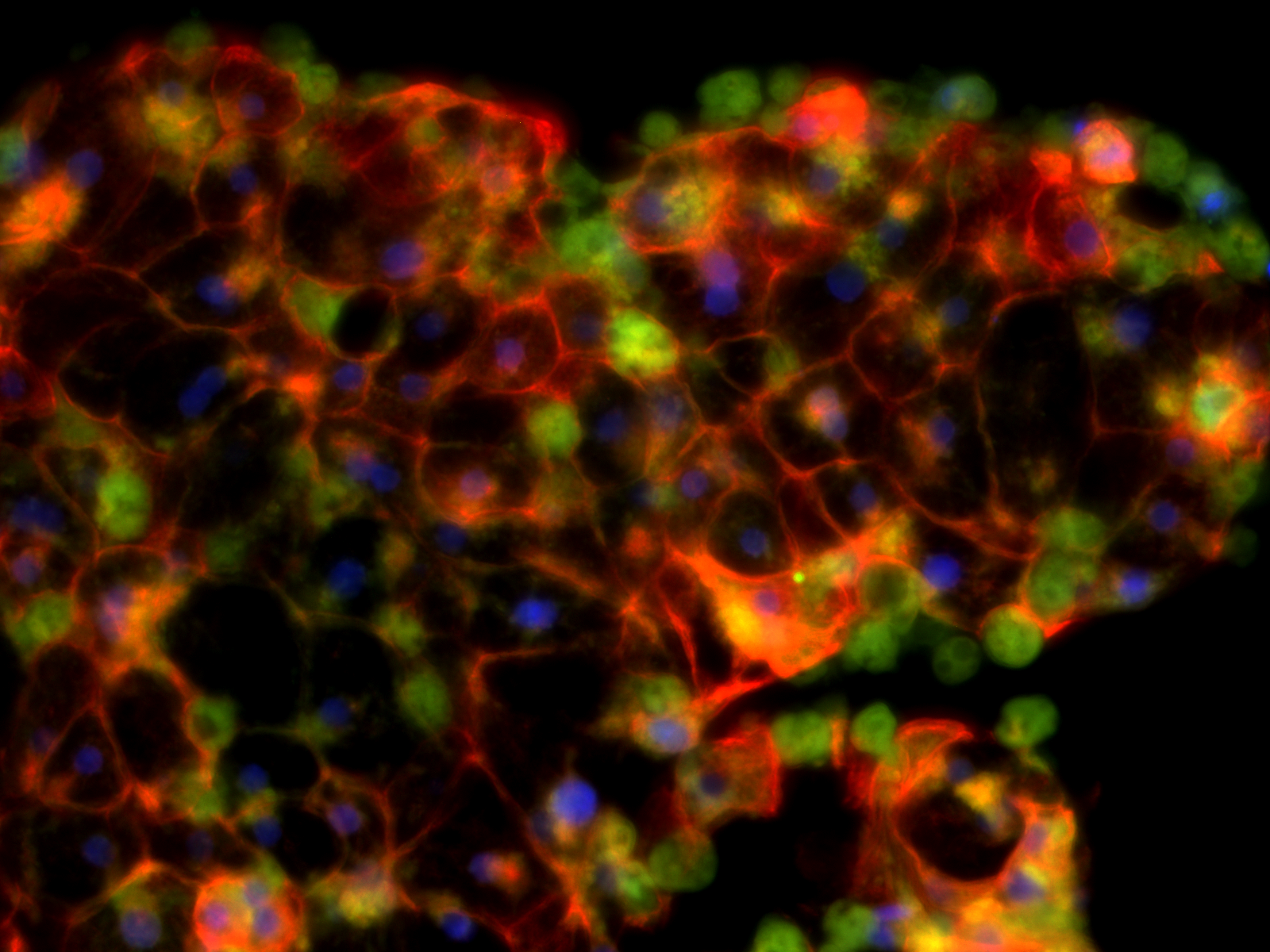

Supplement: Supplementary File 1 [file bioengineering-05-00024-s001.zip › Histology_Immnunofluorescence/CYP1A2_CK18/Control_CYP1A2_CK18_40x_Composite.tif]

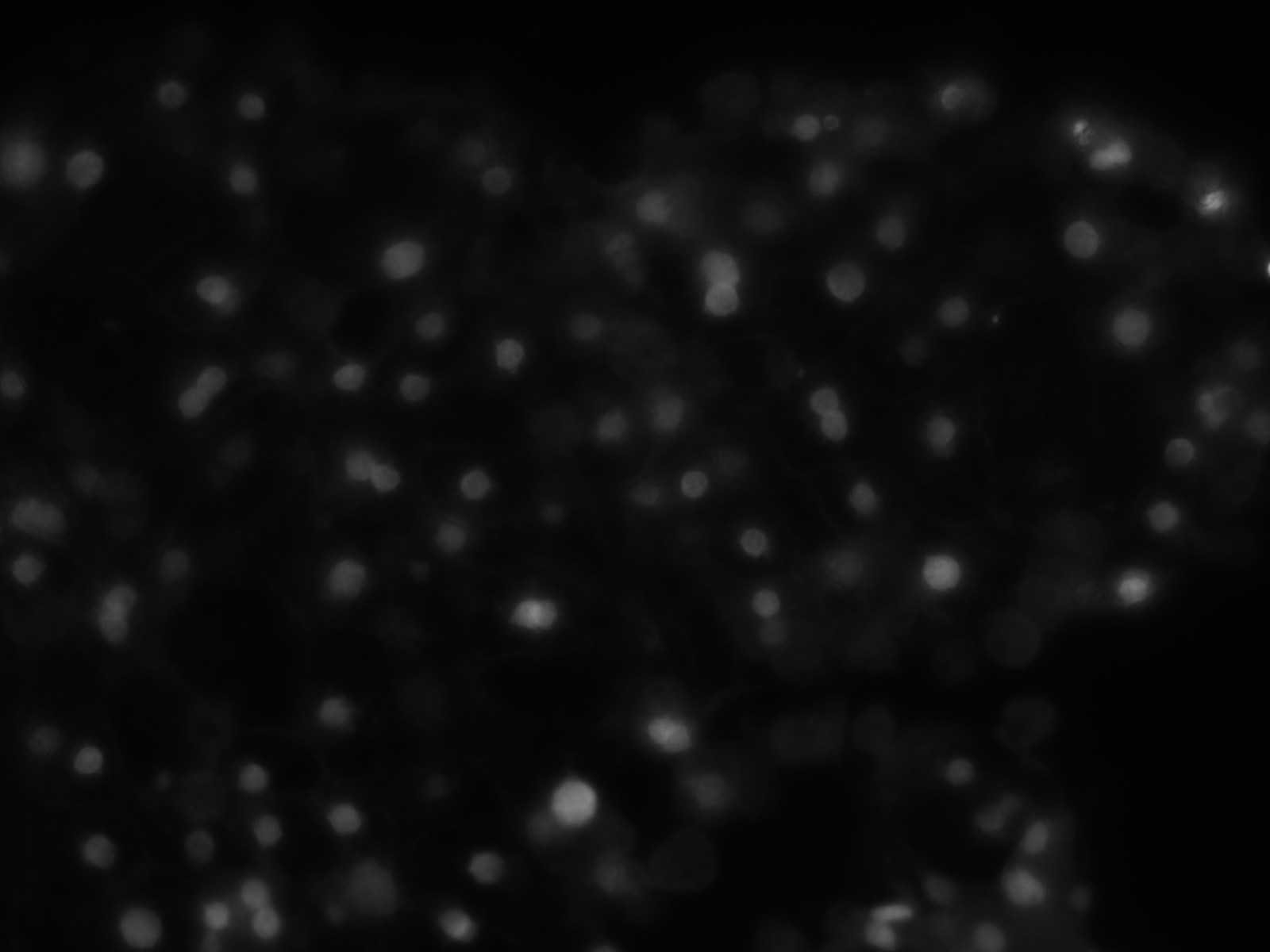

Supplement: Supplementary File 1 [file bioengineering-05-00024-s001.zip › Histology_Immnunofluorescence/CYP1A2_CK18/Control_CYP1A2_CK18_40x_Dapi.tif]

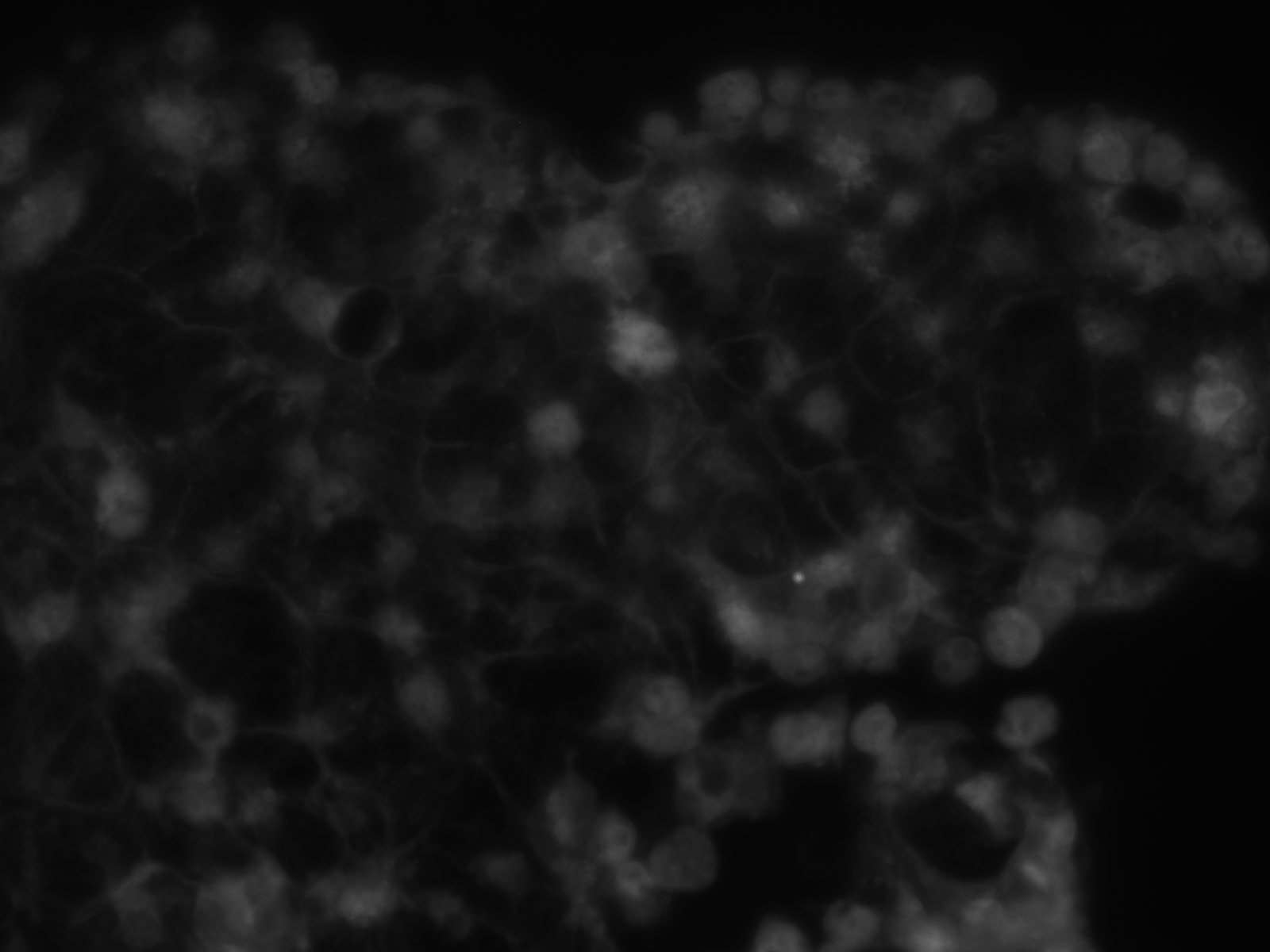

Supplement: Supplementary File 1 [file bioengineering-05-00024-s001.zip › Histology_Immnunofluorescence/CYP1A2_CK18/Control_CYP1A2_CK18_40x_Green.tif]

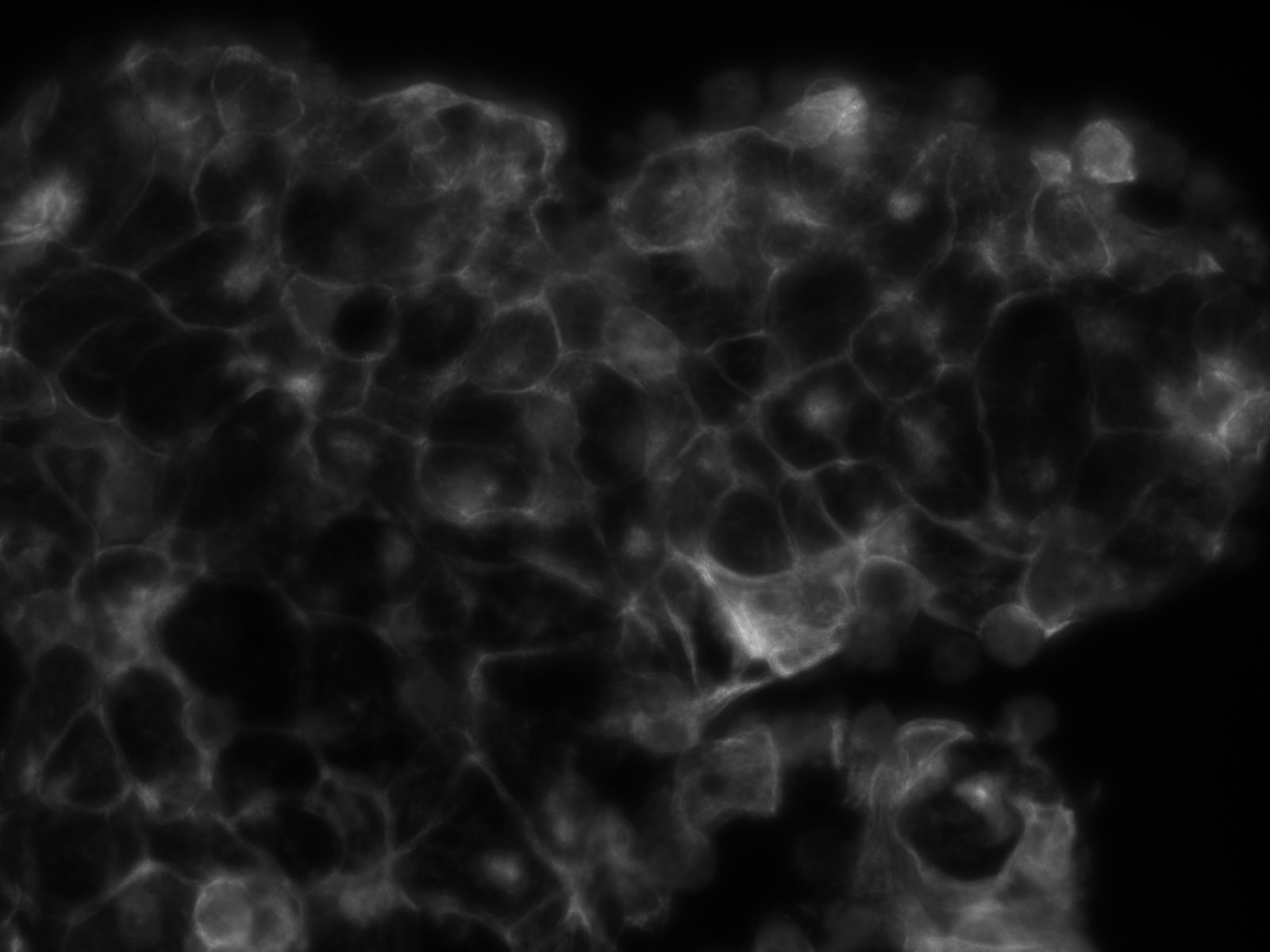

Supplement: Supplementary File 1 [file bioengineering-05-00024-s001.zip › Histology_Immnunofluorescence/CYP1A2_CK18/Control_CYP1A2_CK18_40x_Red.tif]

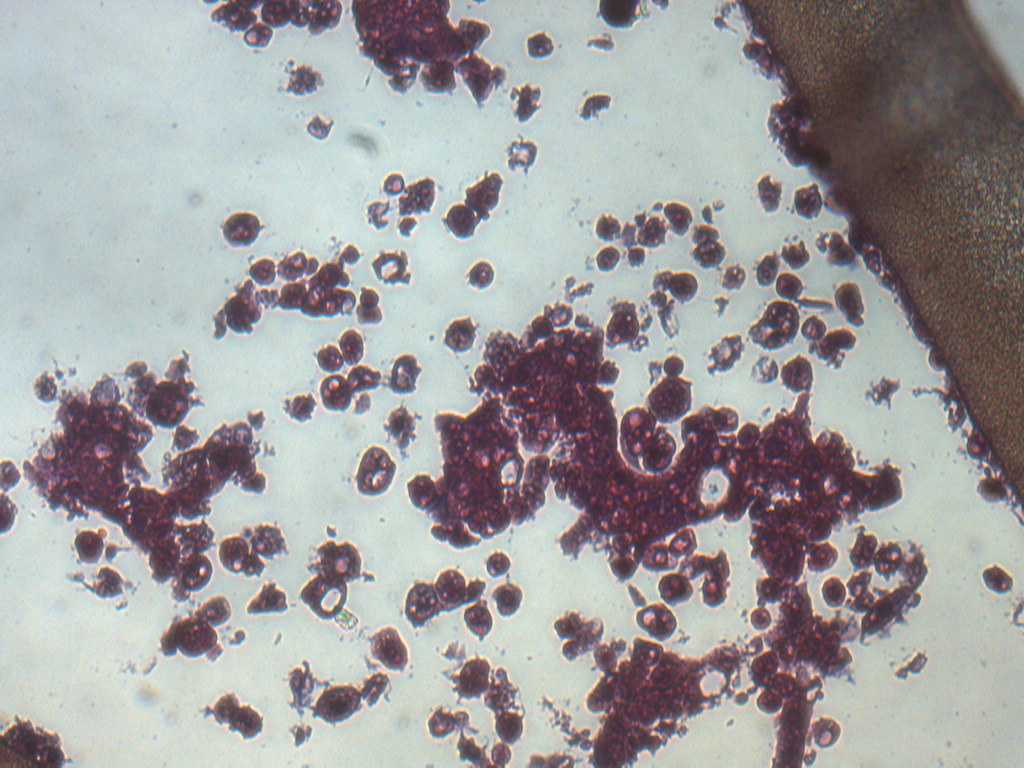

Supplement: Supplementary File 1 [file bioengineering-05-00024-s001.zip › Histology_Immnunofluorescence/H_and_E/10mM_HE_32x.tif]

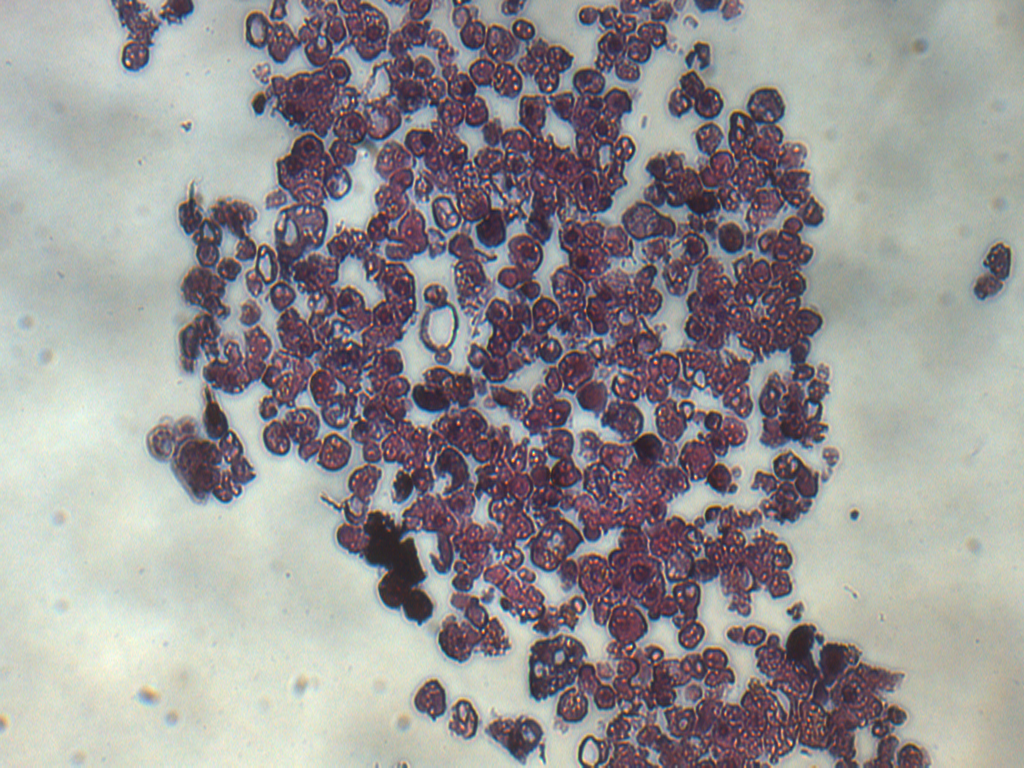

Supplement: Supplementary File 1 [file bioengineering-05-00024-s001.zip › Histology_Immnunofluorescence/H_and_E/30mM_HE_32x.tif]

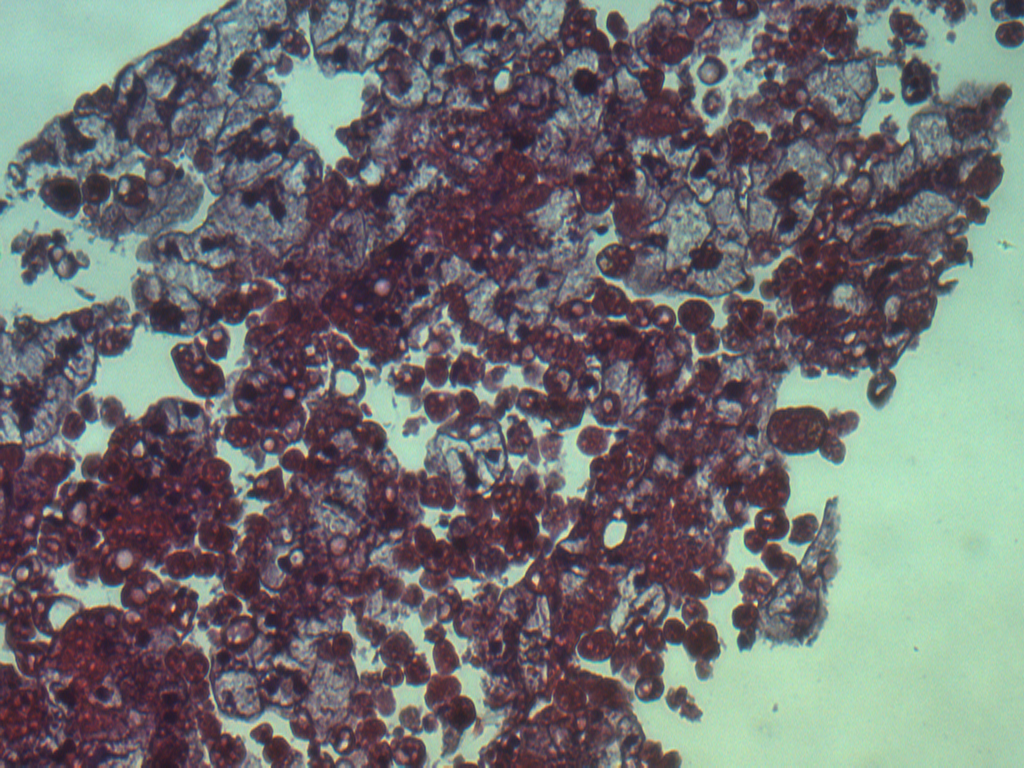

Supplement: Supplementary File 1 [file bioengineering-05-00024-s001.zip › Histology_Immnunofluorescence/H_and_E/Control_HE_32x.tif]

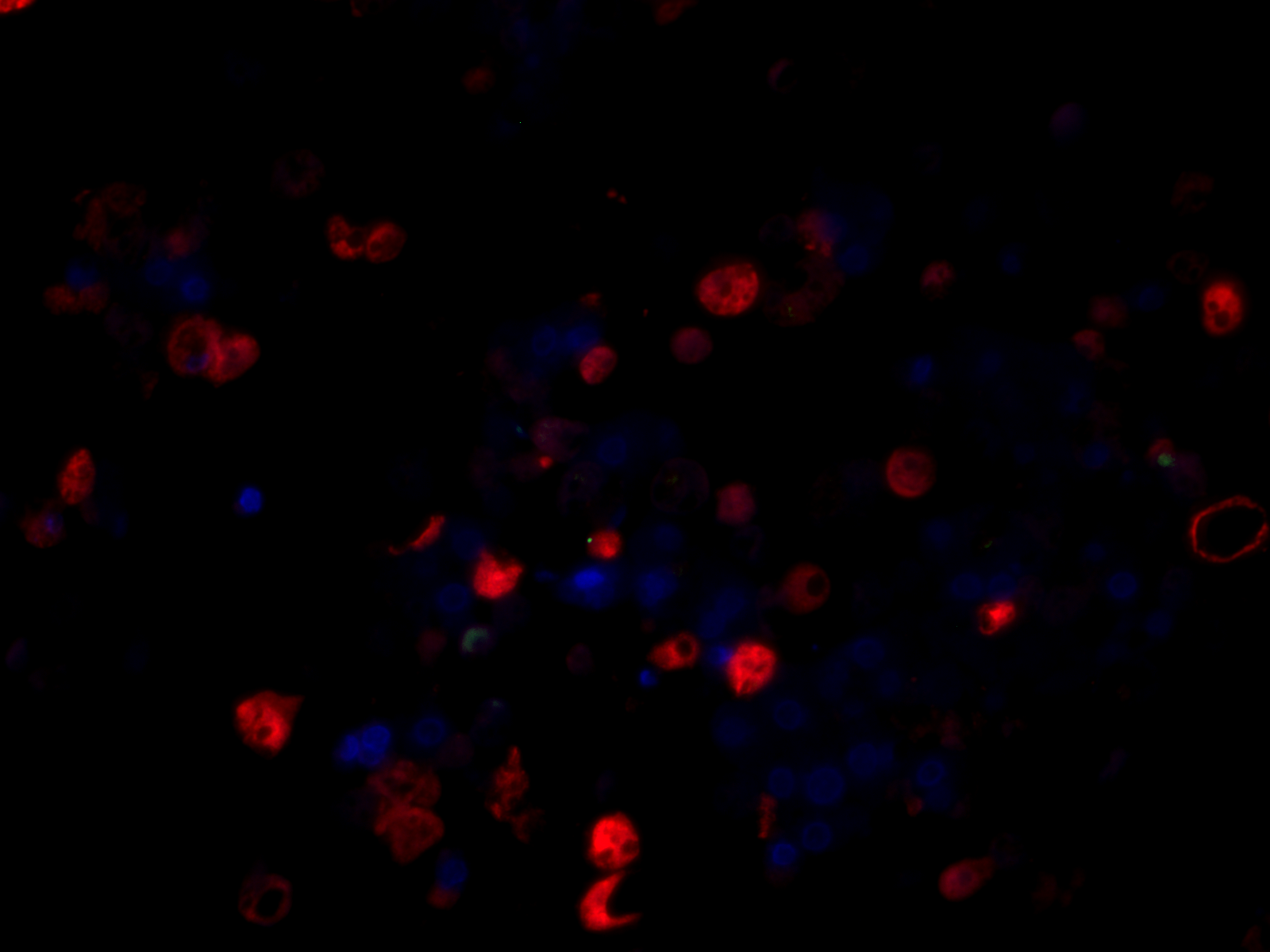

Supplement: Supplementary File 1 [file bioengineering-05-00024-s001.zip › Histology_Immnunofluorescence/MRP2_CYP3A4/10mM_MRP2_CYP3A4_40x_Composite.tif]

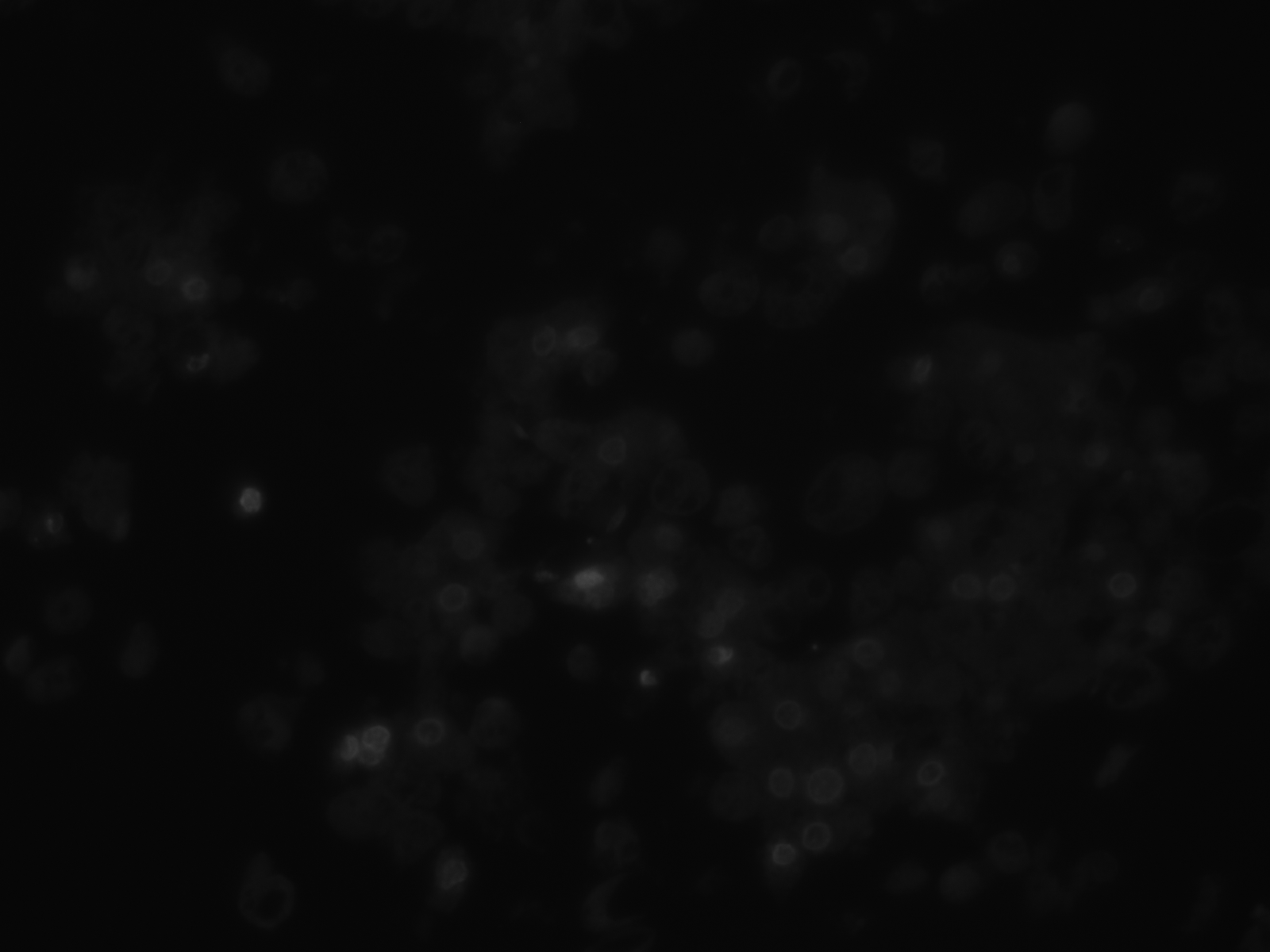

Supplement: Supplementary File 1 [file bioengineering-05-00024-s001.zip › Histology_Immnunofluorescence/MRP2_CYP3A4/10mM_MRP2_CYP3A4_40x_Dapi.tif]

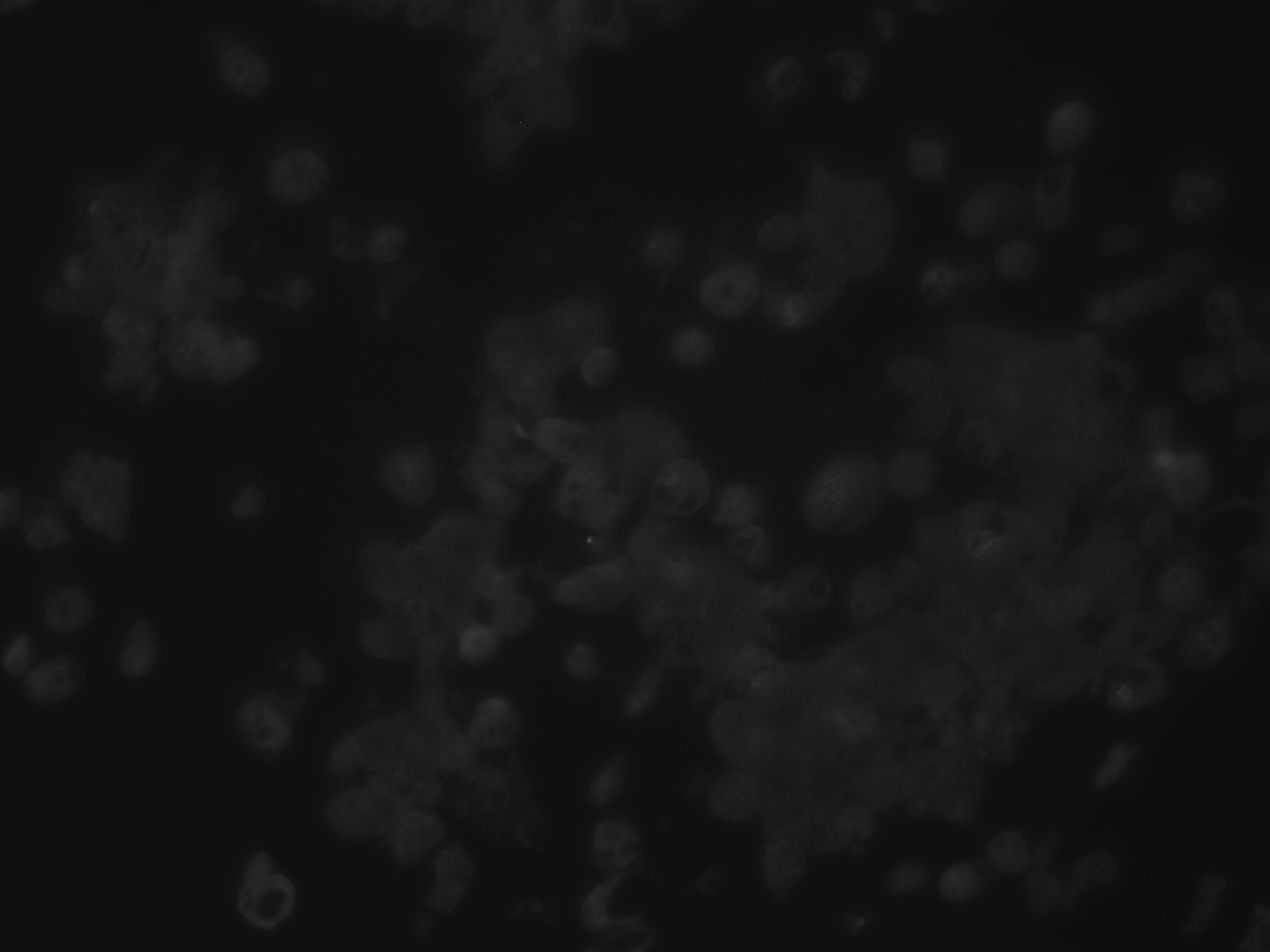

Supplement: Supplementary File 1 [file bioengineering-05-00024-s001.zip › Histology_Immnunofluorescence/MRP2_CYP3A4/10mM_MRP2_CYP3A4_40x_Green.tif]

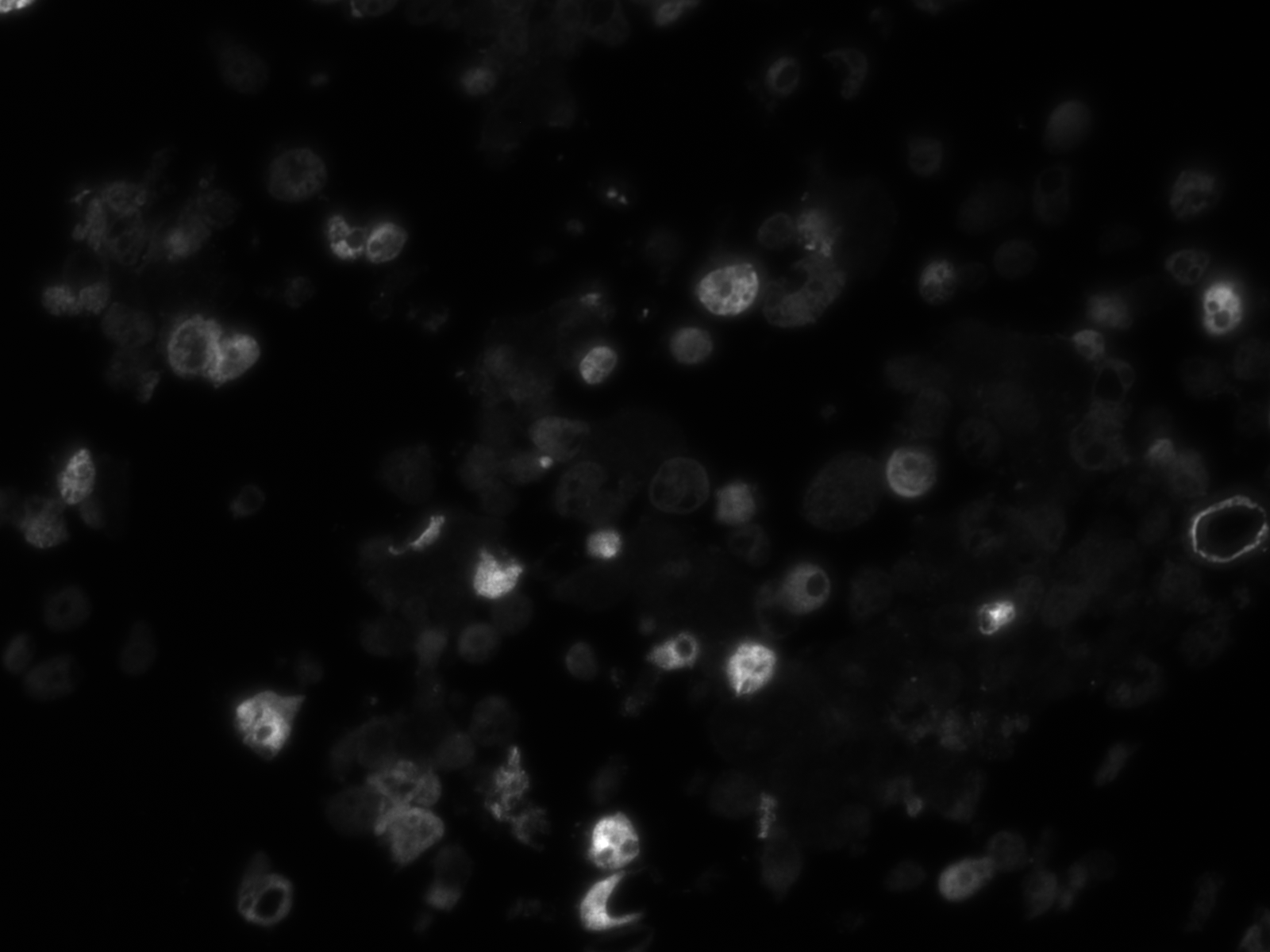

Supplement: Supplementary File 1 [file bioengineering-05-00024-s001.zip › Histology_Immnunofluorescence/MRP2_CYP3A4/10mM_MRP2_CYP3A4_40x_Red.tif]

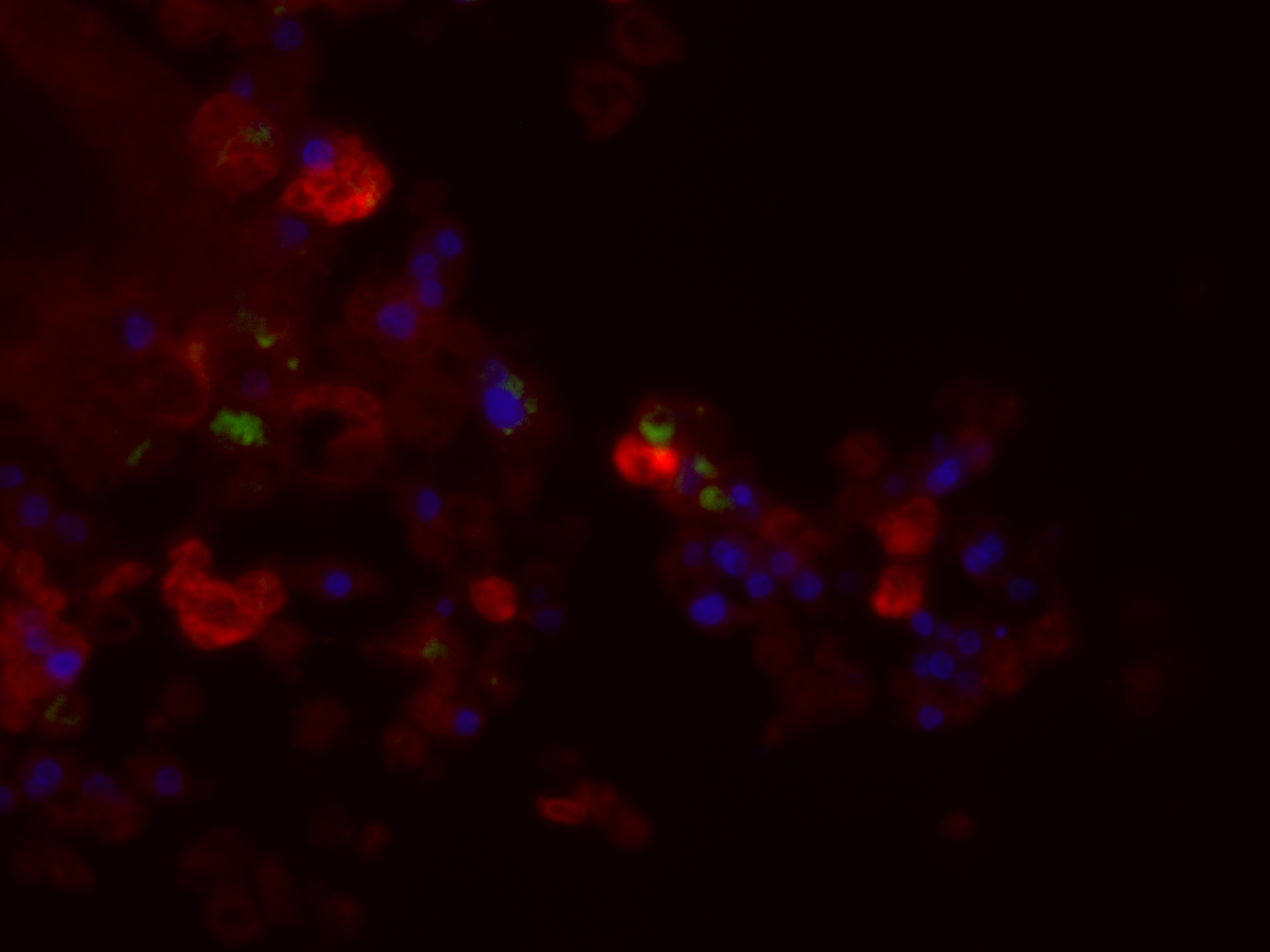

Supplement: Supplementary File 1 [file bioengineering-05-00024-s001.zip › Histology_Immnunofluorescence/MRP2_CYP3A4/30mM_MRP2_CYP3A4_40x_Composite.tif]

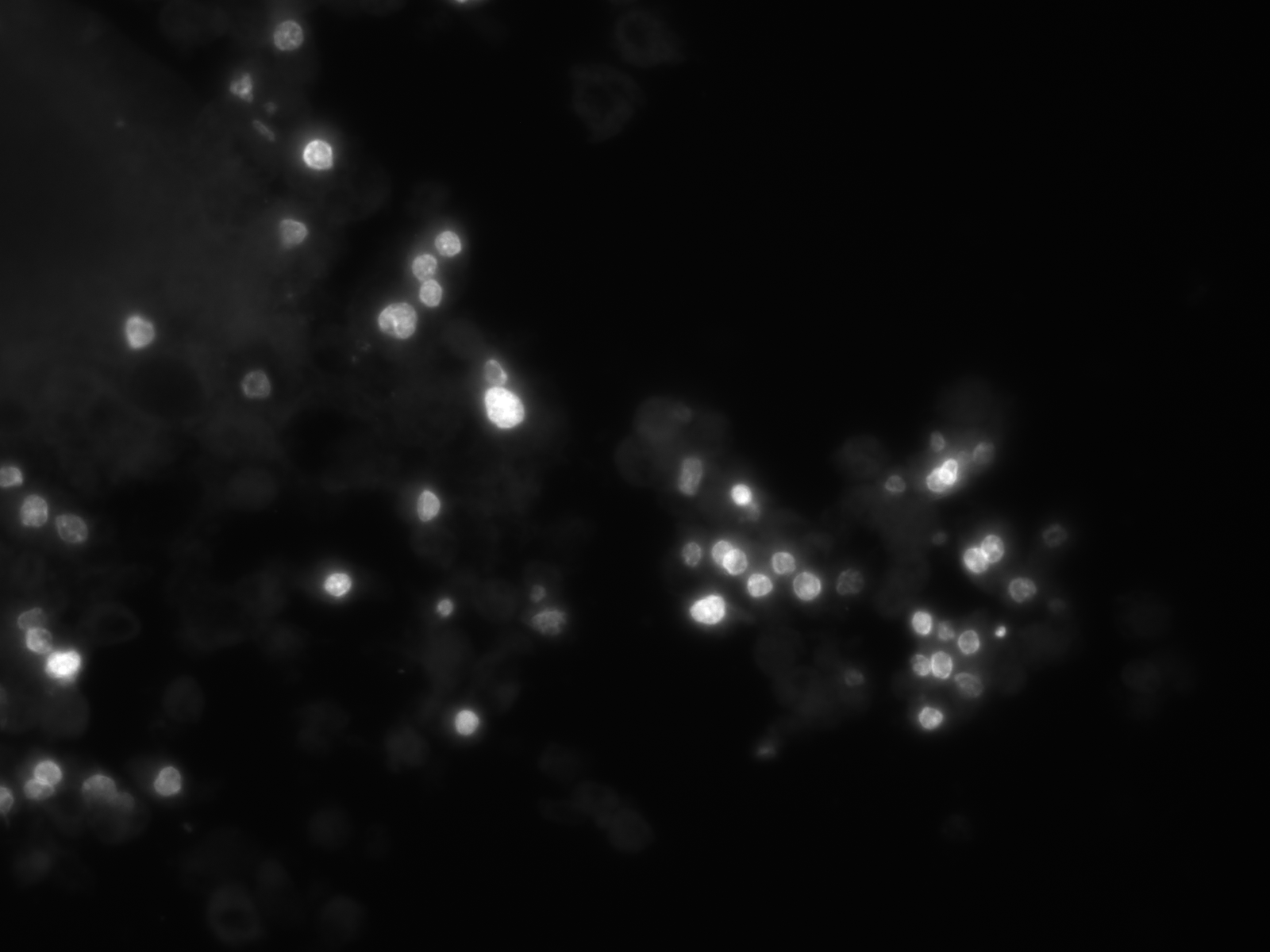

Supplement: Supplementary File 1 [file bioengineering-05-00024-s001.zip › Histology_Immnunofluorescence/MRP2_CYP3A4/30mM_MRP2_CYP3A4_40x_Dapi.tif]

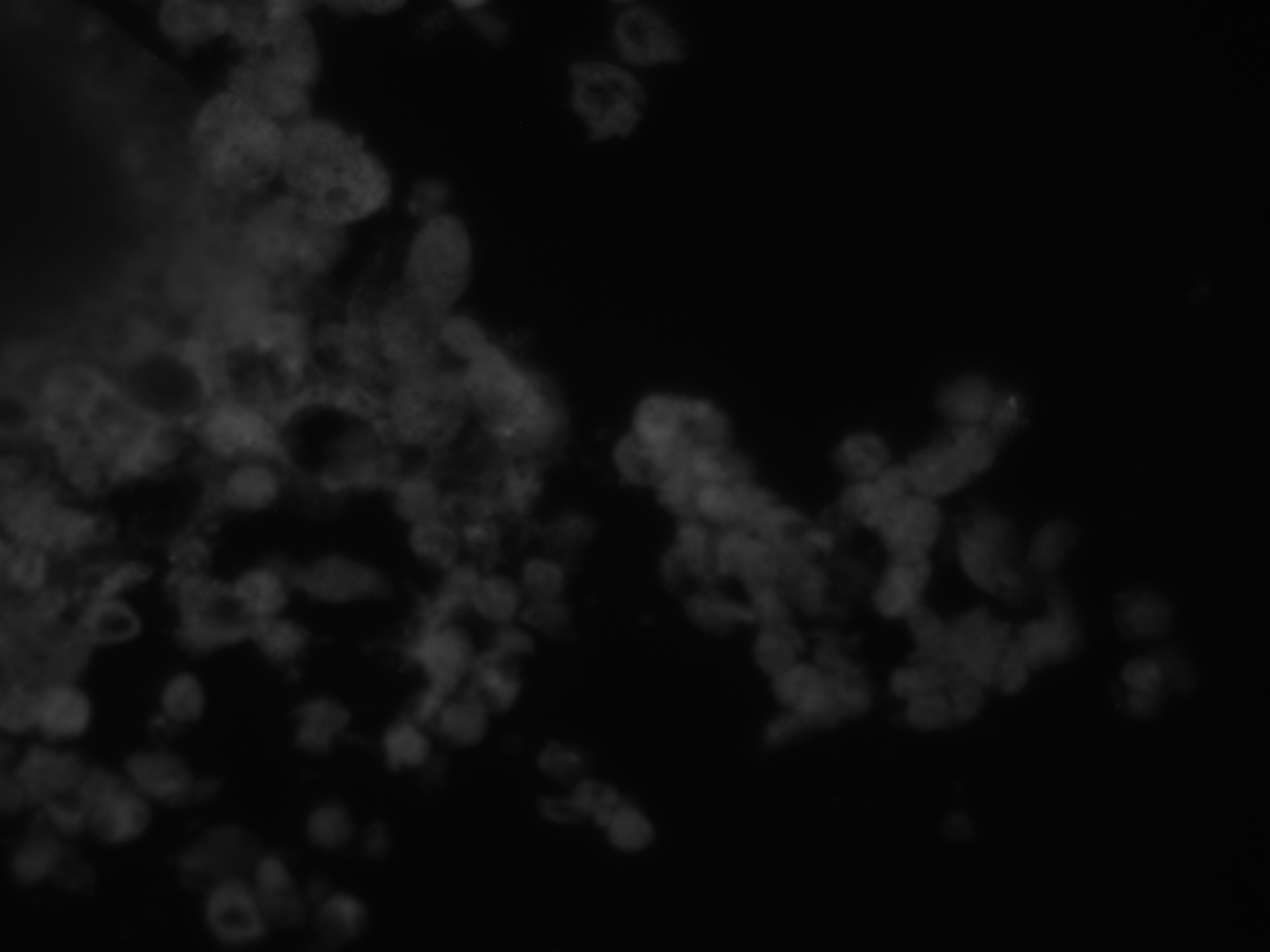

Supplement: Supplementary File 1 [file bioengineering-05-00024-s001.zip › Histology_Immnunofluorescence/MRP2_CYP3A4/30mM_MRP2_CYP3A4_40x_Green.tif]

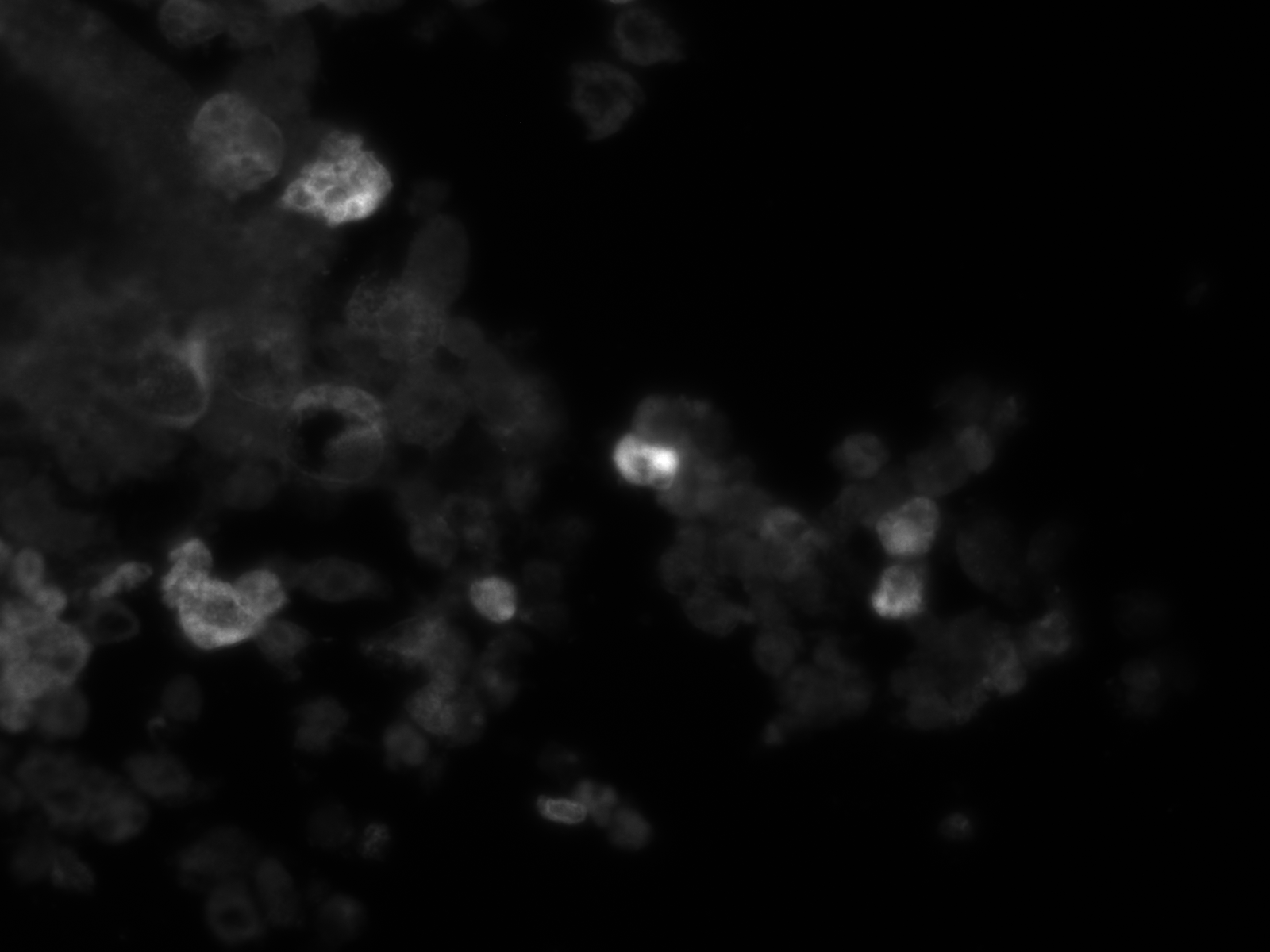

Supplement: Supplementary File 1 [file bioengineering-05-00024-s001.zip › Histology_Immnunofluorescence/MRP2_CYP3A4/30mM_MRP2_CYP3A4_40x_Red.tif]

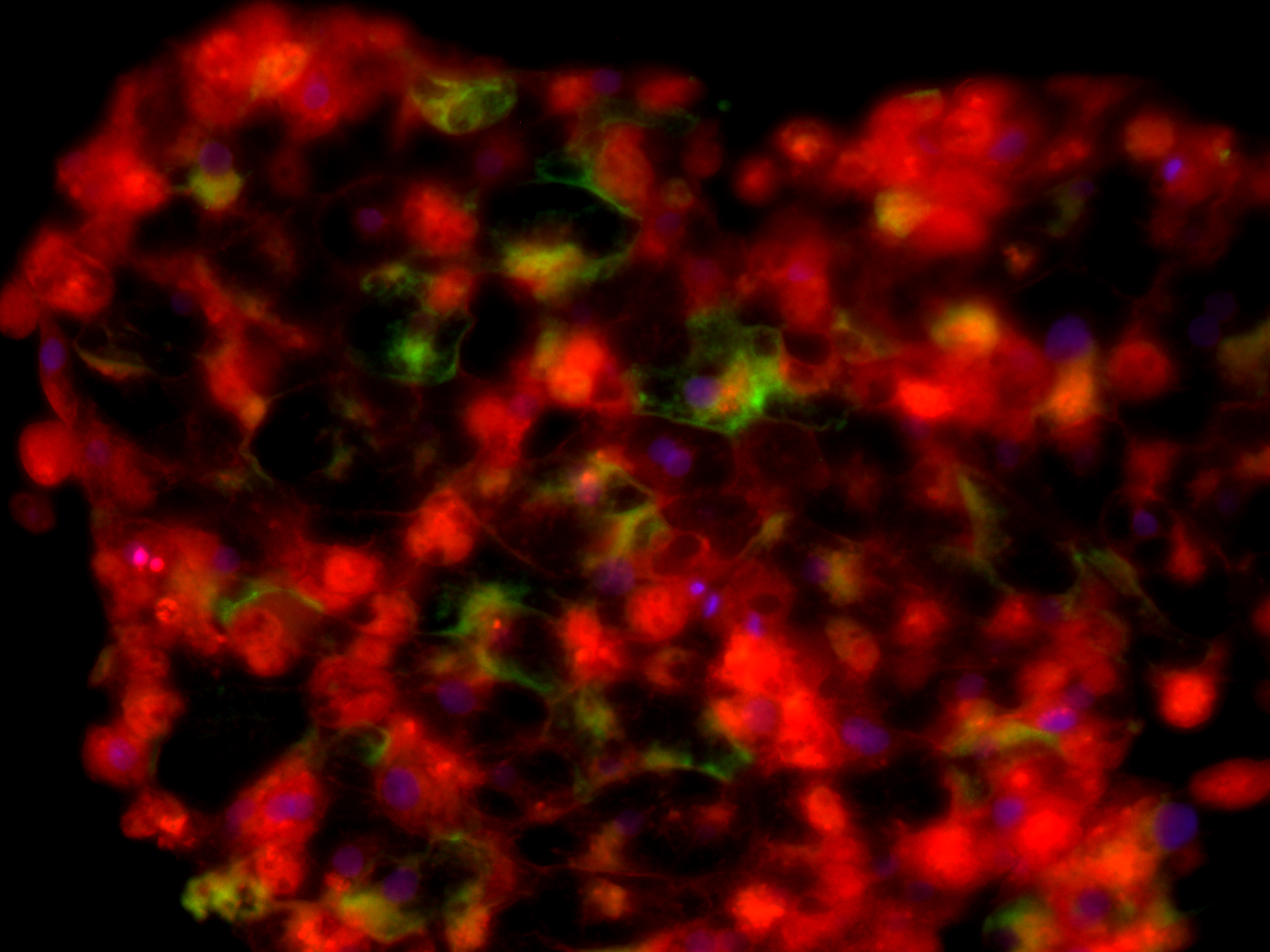

Supplement: Supplementary File 1 [file bioengineering-05-00024-s001.zip › Histology_Immnunofluorescence/MRP2_CYP3A4/Control_MRP2_CYP3A4_40x_Composite.tif]

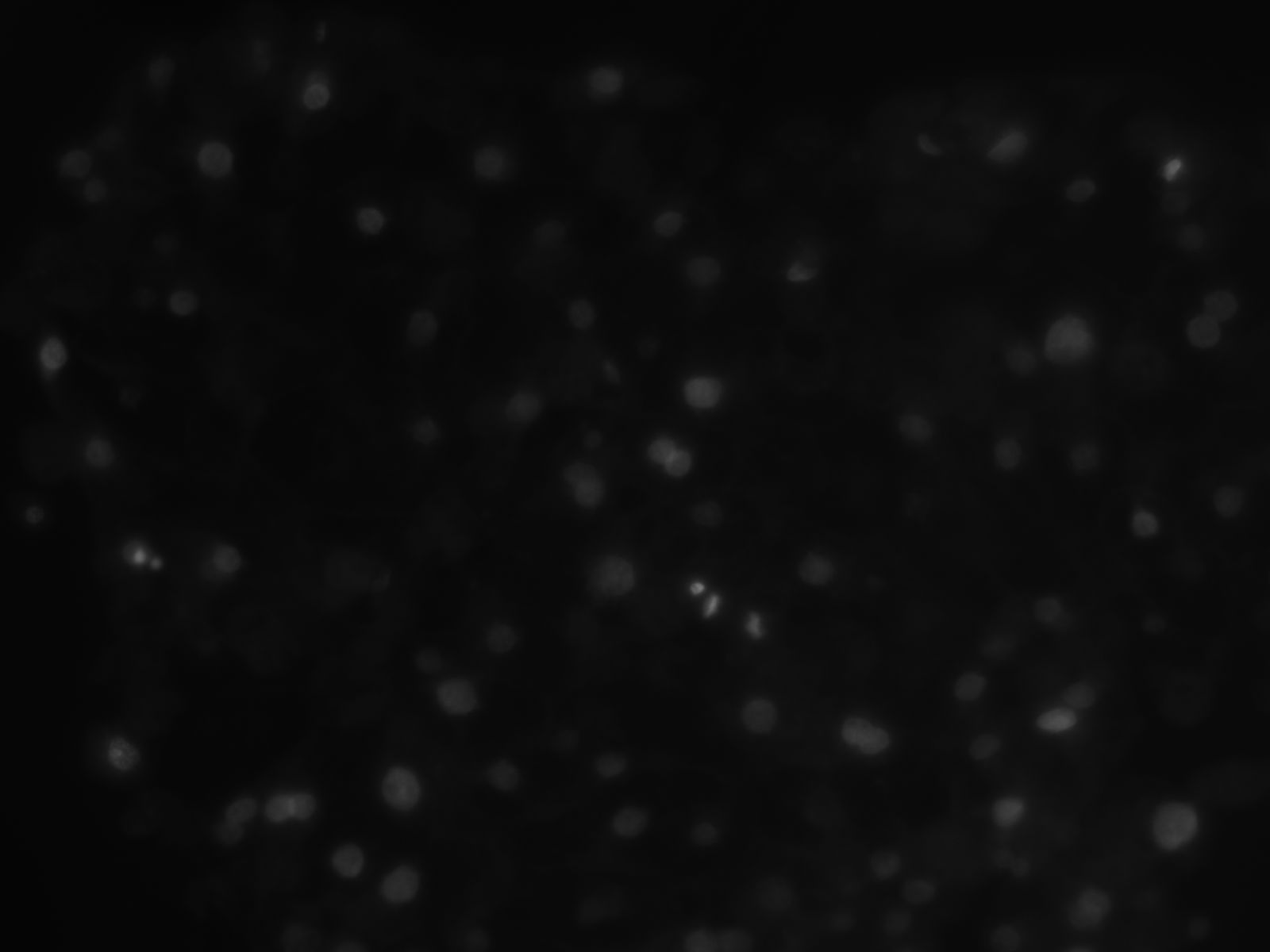

Supplement: Supplementary File 1 [file bioengineering-05-00024-s001.zip › Histology_Immnunofluorescence/MRP2_CYP3A4/Control_MRP2_CYP3A4_40x_Dapi.tif]

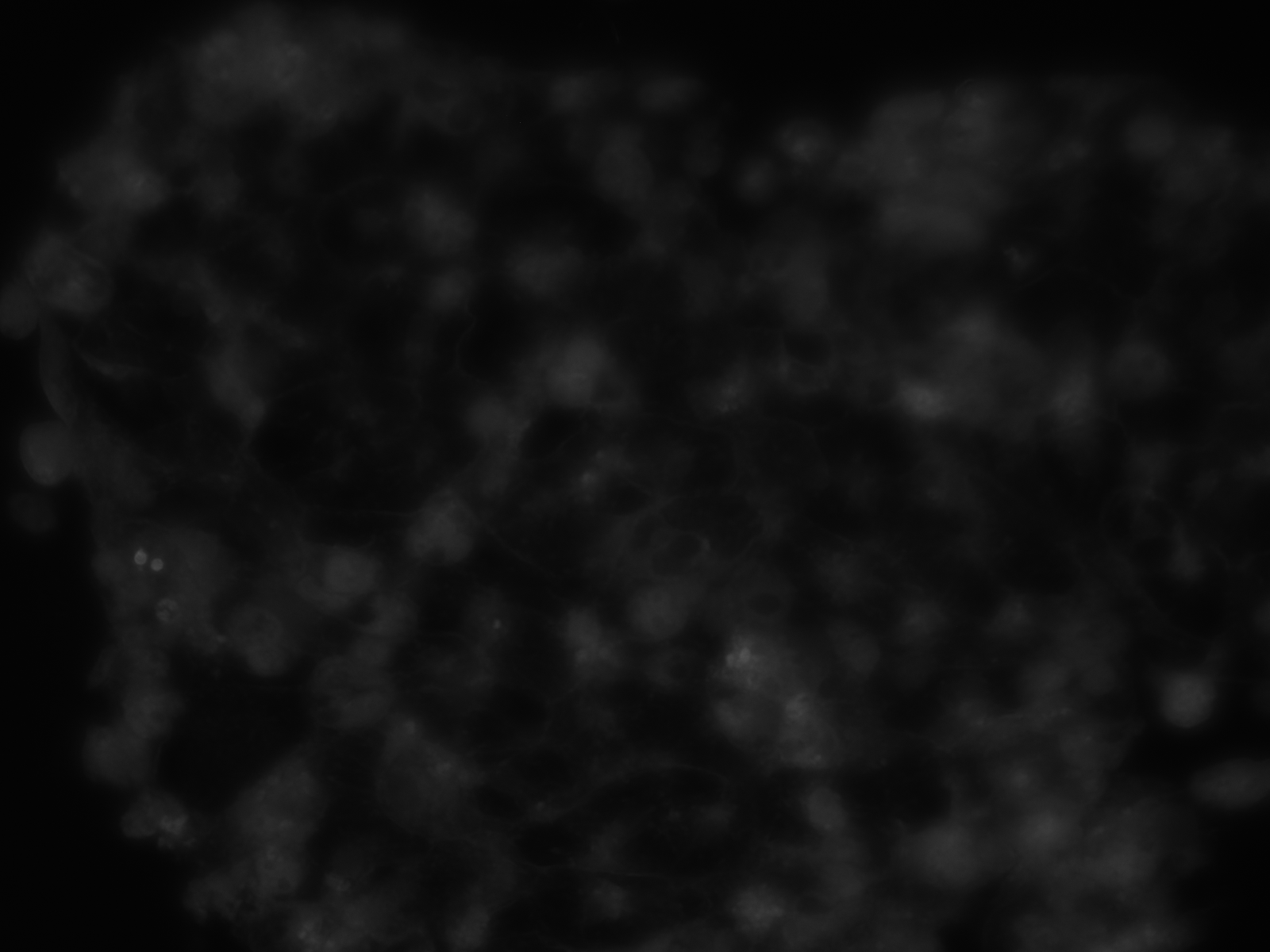

Supplement: Supplementary File 1 [file bioengineering-05-00024-s001.zip › Histology_Immnunofluorescence/MRP2_CYP3A4/Control_MRP2_CYP3A4_40x_Green.tif]

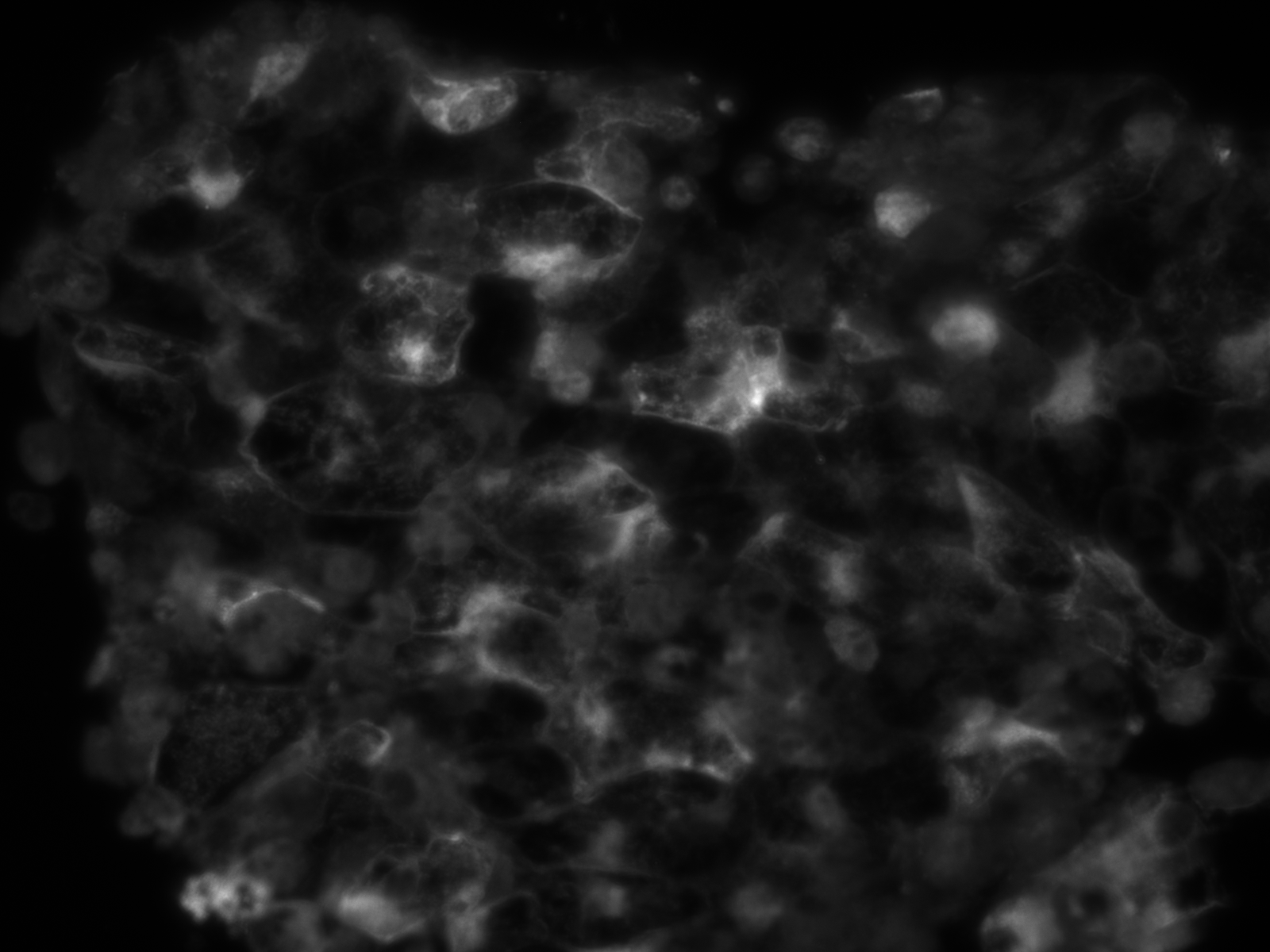

Supplement: Supplementary File 1 [file bioengineering-05-00024-s001.zip › Histology_Immnunofluorescence/MRP2_CYP3A4/Control_MRP2_CYP3A4_40x_Red.tif]
